# Berberine: A Review of its Pharmacokinetics Properties and Therapeutic Potentials in Diverse Vascular Diseases

**DOI:** 10.3389/fphar.2021.762654

**Published:** 2021-11-03

**Authors:** Xiaopeng Ai, Peiling Yu, Lixia Peng, Liuling Luo, Jia Liu, Shengqian Li, Xianrong Lai, Fei Luan, Xianli Meng

**Affiliations:** ^1^ School of Pharmacy, Chengdu University of Traditional Chinese Medicine, Chengdu, China; ^2^ Department of Pharmacy, Affiliated Hospital of North Sichuan Medical College, Nanchong, China; ^3^ School of Ethnic Medicine, Chengdu University of Traditional Chinese Medicine, Chengdu, China; ^4^ Innovative Institute of Chinese Medicine and Pharmacy, Chengdu University of Traditional Chinese Medicine, Chengdu, China

**Keywords:** berberine, cardiovascular disease, cerebrovascular disease, diabetes mellitus, pharmacokinetics

## Abstract

Traditional Chinese medicine plays a significant role in the treatment of various diseases and has attracted increasing attention for clinical applications. Vascular diseases affecting vasculature in the heart, cerebrovascular disease, atherosclerosis, and diabetic complications have compromised quality of life for affected individuals and increase the burden on health care services. Berberine, a naturally occurring isoquinoline alkaloid form *Rhizoma coptidis*, is widely used in China as a folk medicine for its antibacterial and anti-inflammatory properties. Promisingly, an increasing number of studies have identified several cellular and molecular targets for berberine, indicating its potential as an alternative therapeutic strategy for vascular diseases, as well as providing novel evidence that supports the therapeutic potential of berberine to combat vascular diseases. The purpose of this review is to comprehensively and systematically describe the evidence for berberine as a therapeutic agent in vascular diseases, including its pharmacological effects, molecular mechanisms, and pharmacokinetics. According to data published so far, berberine shows remarkable anti-inflammatory, antioxidant, antiapoptotic, and antiautophagic activity via the regulation of multiple signaling pathways, including AMP-activated protein kinase (AMPK), nuclear factor κB (NF-κB), mitogen-activated protein kinase silent information regulator 1 (SIRT-1), hypoxia-inducible factor 1α (HIF-1α), vascular endothelial growth factor phosphoinositide 3-kinase (PI3K), protein kinase B (Akt), janus kinase 2 (JAK-2), Ca^2+^ channels, and endoplasmic reticulum stress. Moreover, we discuss the existing limitations of berberine in the treatment of vascular diseases, and give corresponding measures. In addition, we propose some research perspectives and challenges, and provide a solid evidence base from which further studies can excavate novel effective drugs from Chinese medicine monomers.

## Introduction

The global health burden of vascular diseases, such as atherosclerosis, cerebrovascular disease, hypertension, and complications of diabetes, is rapidly increasing (Al Rifai et al., 2021; Ji et al., 2021; Riccardi et al., 2021). Epidemiological surveys have shown that the increasing cost of vascular diseases worldwide compromises quality of life for individuals (Liss et al., 2021). In addition, a broad variety of factors, including inflammation, vascular dysplasia, oxidative stress, and abnormal lipid metabolism, cause vascular diseases (Guzik and Touyz, 2017; Feng et al., 2020). Hence, strategies aiming to reduce inflammation and oxidative stress and normalize the lipid metabolism are generally used to treat and prevent the vascular diseases, and statins, nonsteroidal anti-inflammatory drugs, and novel biological agents are common therapeutic agents (Oesterle et al., 2017; Lu et al., 2018; Doña et al., 2020). However, the high cost and side effect profiles of these drugs make finding cheaper alternatives with fewer side effects and similar or better therapeutic outcomes a matter of urgency. Therapies used in traditional Chinese medicines (TCM) have long been used as complementary and alternative medicines for the treatment of vascular disease in China (Cheng et al., 2017; Li et al., 2018b). Recently, these have garnered research interest owing to fewer adverse reactions and lower toxicities of these compounds compared with those identified and used in western medicine (Xie et al., 2019; Oduro et al., 2020; Atanasov et al., 2021). Undeniably, TCM has made an indelible contribution to human health and is considered a potential source of therapies derived from natural, rather than synthetic, sources. Therefore, there is an increased emphasis on the use of medicinal plants such as those used in TCM in the development of novel drugs.

Berberine (C_20_H_18_NO_4_^+^, CAS no: 2086–83-1, Figure 1), a naturally occurring benzylisoquinoline alkaloid, has a long history of medical applications in TCM (Li et al., 2019). As a natural bioactive ingredient, berberine mainly exists naturally in the roots, rhizomes, and stem bark of various medicinal plants from the Ranunculaceae (Wang et al., 2019), Rutaceae (Ryuk et al., 2012), and Berberidaceae families (Gawel et al., 2020). Berberine was reportedly used in China as a folk medicine by Shennong at approximately 3000 BC, and the first recorded use of berberine is described in the ancient Chinese medical book The Divine Farmer’s Herb-Root Classic (Neag et al., 2018). The hydrochloride salt of berberine, listed as an oral antibacterial agent in Pharmacopoeia of the People’s Republic of China, is a common over-the-counter medication; dosage is usually 0.1 g in pill form taken 1–3 times per day for gastrointestinal infections (Zhang et al., 2021b). Colloquially, it is known as Huangliansu (Chinese: 黄连素; literally translated into English: “the essence of Chinese goldthread”). Modern pharmacological studies have confirmed that berberine exhibits various clinically useful biological properties, including anticardiovascular disease and anticancer properties (Feng et al., 2019; Hu et al., 2019). A growing body of evidence has shown that berberine has poor bioavailability due to first-pass effects in the intestinal lumen, leading to limitations in its clinical application (Xu et al., 2019; Habtemariam, 2020b). However, berberine is currently being evaluated in clinical trials for its important clinical benefits, lower toxicity and side effects compared with currently available therapies in western medicine, with its active metabolites exerting similar bioactive properties as berberine itself (Kumar et al., 2015; Imenshahidi and Hosseinzadeh, 2019). Further studies on berberine’s mechanism of action as well as new applications and novel formulations are therefore warranted.

**FIGURE 1:**
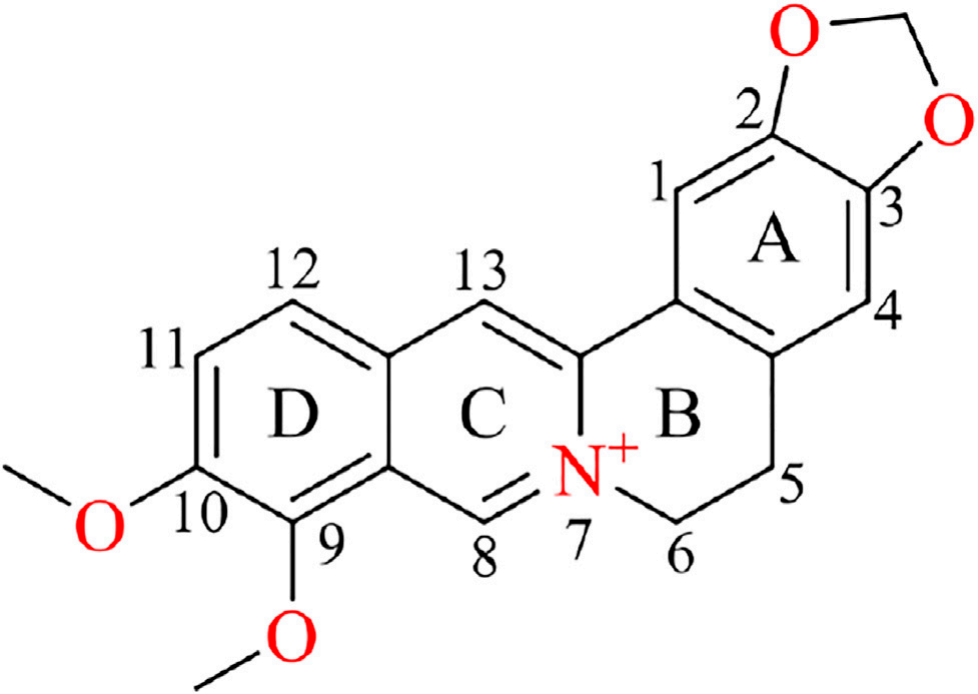
Schematic representation of the chemical structure of berberine.

In recent years, novel unique molecular entities derived from herbal medicines containing berberine have led to increased attention to the potential of this compound in the treatment of vascular diseases (Lee et al., 2018; Ren et al., 2019; Rajabi et al., 2021). Along with advances in pharmacological research, berberine was considered one of the most promising naturally derived drugs for the treatment of numerous human vascular diseases through the modulation of multiple signaling pathways. However, no systematic reviews on the pharmacological and pharmacokinetic properties of berberine in the context of vascular disease have been published. Therefore, in this review, we screened articles on berberine treatment in vascular diseases published in the years 2010–2021 using Web of Science, ScienceDirect, PubMed, Google Scholar and China National Knowledge Infrastructure online databases and summarized the findings to provide insights into the potential application of berberine in vascular diseases.

## Physical and Chemical Properties of Berberine

Berberine is a yellow solid, with a melting point of 145.1–146.7°C; it is soluble in hot water, slightly soluble in cold water or ethanol, and insoluble in benzene, ether, chloroform, and other organic solvents (Zhang et al., 2016a). The structure of berberine comprises a dihydroisoquinoline ring and an isoquinoline ring with planar characteristics (Figure 1). The skeleton can be divided into four rings, A, B, C, and D, with the C_2_ and C_3_ of the A ring forming a methylenedioxy group responsible for most of the biological activities of berberine, such as anticancer activity (Leyva-Peralta et al., 2019). The “C” ring contains a quaternary ammonium structure (with N^+^ in the aromatic ring), which is necessary for the antibacterial activity (Gaba et al., 2021). In the “D” ring, C_9_ and C_10_ are each attached to a methoxy group. At present, structural modification studies of berberine mainly focus on the “C” and “D” rings (Xiao et al., 2018; Habtemariam 2020a); available evidence suggests that alkylation or acylation in the “D” ring resulted in hypoglycemic activity (Cheng et al., 2010; Shan et al., 2013). The introduction of cinnamic acid at 9-O position exerted strong hypoglycemic effects (Zhang et al., 2016b). C_8_ and C_13_ alkylation were shown to enhance cytotoxicity (Singh et al., 2021). Similarly, positions N_7_ and C_13_ are prone to modifications that enhance anticellular proliferative activity of berberine (Gaba et al., 2021). Moreover, berberine is fluorescent, with a maximum absorption wavelength of 350 nm and an emission wavelength of 530 nm in 0.01 mol/L sodium dodecyl sulfate solution. Thus, liquid chromatography–mass spectrometry and isotope labeling can be used to measure the content of berberine as part of a TCM or drugs (Chang et al., 2016; Ai et al., 2019).

## Pharmacokinetics of Berberine

### Absorption

Berberine exerts superior therapeutical effects on the vascular diseases, such as atherosclerosis; however, its effects are limited in clinic due to poor oral absorption and low bioavailability (Han et al., 2021b). Previous research has reported that the absolute bioavailability of berberine is 0.37% when administered in a single oral administration (48.2, 120, or 240 mg/kg body weight) in rats (Feng et al., 2021). Oral treatment with 100 mg/kg berberine has an absolute bioavailability of 0.68% as measured in rat plasma samples, with a mean maximum plasma concentration (C_max_) of 9.48 ng/ml and an area under the curve (AUC)_0–36 h_ of 46.5 ng h/ml (Chen et al., 2011). Sahibzada et al. (2021) found that after a single oral dose of 50 mg/kg berberine in rabbits, the C_max_ was 0.411 μg/ml.

Some studies have also reported the absorption of berberine in humans. In one study, the mean C_max_ at 8 h post-administration was reported to be approximately 0.4 ng/ml for 400 mg berberine administered orally (*n* = 20) (Hua et al., 2007). Another study reported that the C_max_ of berberine in 10 healthy individuals given 500 mg berberine orally was extremely low, at 0.07 nM (Spinozzi et al., 2014). It is thought that the lower *in vivo* bioavailability of berberine is closely related to extensive intestinal first-pass elimination, in which the drug is filtered out of the circulation by the liver resulting in a low level of systemic circulation (Guan et al., 2018). After orally administrated with 100 mg/kg berberine to rats, approximately half of berberine ran intact through the gastrointestinal tract and another half was disposed of by the small intestine, resulting in an extremely low extent of absolute oral bioavailability (0.36%) (Liu et al., 2010b). Additionally, a caco-2 cell monolayer model was used to confirm that berberine is the substrate for the drug transporter P glycoprotein, which may contribute to the lower absorption of berberine in small intestinal epithelial cells by passive diffusion (Zhang et al., 2011; Cui et al., 2015).

With its remarkable pharmacological activity, berberine has been used for a variety of diseases in the clinic. However, due to its low *in vivo* bioavailability, exploring methods that increase the concentration of berberine in blood is key to improving its usefulness in this context. Although intravenous administration provides a direct approach that may improve the bioavailability of berberine, this can lead to serious side effects including respiratory arrest (Han et al., 2021b). Therefore, berberine is often administered orally in clinic. Conversion of biological small molecules into salt compounds may be a method to improve its bioavailability *in vivo*. The bioavailability of berberine organic acid salts, especially berberine fumarate and berberine succinate, is higher than that of berberine hydrochloride (Cui et al., 2018). Moreover, chemical structure modification can be used to improve bioavailability of this drug. Long-chain alkylation (C_5_-C_9_) may enhance hydrophobicity, which has been shown to improve bioavailability; for example, 9-O-benzylation further enhances lipophilicity and imparts neuroprotective effect (Lin et al., 2020; Singh et al., 2021).

### Distribution

It has been demonstrated that berberine is rapidly distributed through tissues in the liver, kidneys, muscle, lungs, brain, heart, pancreas, and fat, in descending order of amount, while the concentration of berberine in most of these tissues was higher than that in plasma 4 h after oral administration at a dose of 200 mg/kg in rats. Moreover, berberine concentrations remained relatively stable in liver, heart, brain, muscle, and pancreas tissue in rats (Tan et al., 2013).

However, recent studies on the distribution of berberine *in vivo* are rare, which may be attributed to the broad tissue distribution *in vivo* after oral administration. The availability of new technologies such as component analysis by high-performance liquid chromatography electrospray ionization mass spectrometry (HPLC–ESIMS)/mass spectrometry (MS) and MS imaging may permit improved exploration of the berberine tissue distribution (Jove et al., 2019). The fact that berberine is widely distributed in tissues may be useful in the treatment of some diseases, which may broaden the scope of its clinical application. For example, with the character of enrichment in the liver, oral treatment with 100 mg/kg berberine may promote the excretion of cholesterol from the liver to the bile (Li et al., 2015b). Thus, distribution of berberine may be an important pharmacokinetic property requiring further study in future.

### Metabolism

One study used a sensitive HPLC-ESIMS/MS method to identify the metabolites of berberine in human plasma, of which berberrubine was most abundant, with high lipid solubility in individuals who received 15 mg/kg oral berberine chloride per day for 3 months (Spinozzi et al., 2014). Evidence showed that berberine had a similar metabolic profile in rats (100 mg/kg administered orally) and humans (300 mg administered orally three times a day for 2 days) via the urine (Qiu et al., 2008). Using liquid chromatography coupled with ion trap time-of-flight mass spectrometry, Ma et al. (2013) revealed that 16 separate metabolites could be identified in rat bile, urine, and feces samples after oral administration of berberine (200 mg/kg). After a single oral administration (48.2, 120, or 240 mg/kg) of berberine in rats, the levels of phase 2 metabolites were much higher than those of phase 1 metabolites for the AUC_0–48 h_ values. Simultaneously, nine major metabolites of berberine (demethyleneberberine, jatrorrhizine-3-O-β-D-glucuronide, jatrorrhizine, berberrubine-9-O-β-D-glucuronide, jatrorrhizine-3-O-sulfate, berberrubine, thalfendine-10-O-β-D-glucuronide, demethyleneberberine-2-O-sulfate, and demethyleneberberine-2-O-β-D-glucuronide) were detected in rat serum using a LC–MS/MS method (Feng et al., 2021). Additionally, it was demonstrated that the metabolism of berberine by oral is closely related to liver function and gut microbiota. After oral administration of 300 mg/kg berberine in mice, cytochrome P3A11 (CYP3A11) and CYP3A25 mRNA and CYP3A11 and CYP2D22 enzyme activity levels were all found to be decreased, while the level of CYP1A2 mRNA was increased (Guo et al., 2011). Similarly, on oral administration of 200 mg/kg berberine in rats, the drug was shown to be metabolized in the liver by the CYP450 isoenzyme via oxidative demethylation at C_2_, C_3_, C_9,_ and C_10_, followed by conjugation of the hydroxyl groups with glucuronic acid (Singh et al., 2021). Furthermore, gut microbiota can also affect the metabolism of berberine after oral administration. It was demonstrated that 200 mg/kg berberine administered orally could be converted into absorbable dihydroberberine by nitroreductases produced by gut microbiota, which showed a nearly 5-fold higher intestinal absorption rate than berberine in rats; the dihydroberberine is then oxidized back to berberine after absorption into the intestinal tissue, and enters the blood (Feng et al., 2015; Han et al., 2021a). Also, gut microbiota was shown to convert berberine into oxyberberine through an oxidation reaction *in vitro* and *in vivo*, which exerted a much stronger binding interaction with hemoglobin than plasma (Li et al., 2020a; Chen et al., 2021).

To summarize this section, the liver and intestine are the main metabolizing organs of berberine by oral administration. Inhibiting the first-pass effect may reduce the metabolism of berberine and improve its bioavailability. Interestingly, according to an in-depth study on the metabolism of berberine *in vivo*, it found that phase II metabolites are the major metabolic products of berberine (Feng et al., 2021), whereas the opposite was true in previous studies (Ma et al., 2013). In addition, particular attention should be paid to nitroreductases produced by gut microbiota, and berberine metabolism in general, in future studies, in order to fully establish the pharmacodynamic basis of this TCM.

### Excretion

To better understand the poor absorption of berberine *in vivo*, some researchers have paid more attention to the excretion of berberine via the digestive tract. Berberine was found in feces with a recovery rate of 22.74% after a single oral dose (200 mg/kg) in 48 h, and thalifendine was the most abundant berberine metabolite excreted in the bile, urine, and feces in rats (Ma et al., 2013). In another study, 18.6% of the berberine was excreted in feces as berberrubine after intragastric administration at a single dose of 48.2 mg/kg. The total recovery of berberine and its metabolites from the urine, bile, and feces was 41.2% in rats (Feng et al., 2021). To summarize, berberine and its metabolites are mainly excreted by the kidneys (urine and feces) and bile in rats and mice (Liu et al., 2016).

## Effects of Berberine on Vascular Diseases

**FIGURE 2:**
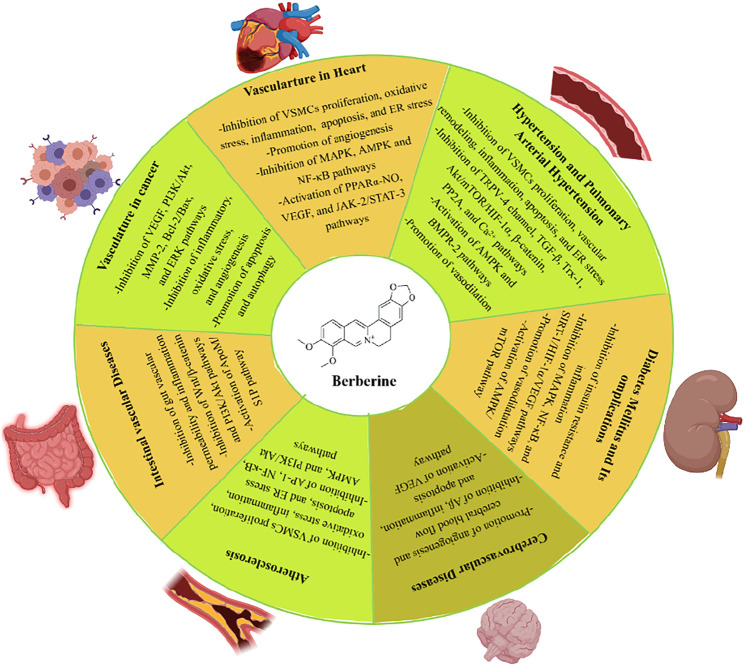
Biological activities and potential pathways of berberine on vascular diseases. Abbreviations: Akt, protein kinase B; AMPK, AMP-activated protein kinase; AP-1, activator protein 1; Bax, Bcl-2 associated X protein; Bcl-2, B-cell lymphoma 2; BMPR-2, bone morphogenetic protein type 2; ER, endoplasmic reticulum; ERK, extracellular signal-regulated kinase; HIF-1α, hypoxia-inducible factor 1α; JAK-2, janus kinase 2; MAPK, mitogen-activated protein kinase; MMP-2, matrix metalloprotease 2; NF-κB, nuclear factor κB; PI3K, phosphoinositide 3-kinase; SIRT-1, silent information regulator 1; STAT-3, signal transducer and activator of transcription 3; TGF-β, transforming growth factor β; TRPV-4, transient receptor potential vanilloid 4; VEGF, vascular endothelial growth factor; VSMCs, vascular smooth muscle cells.

Recently, vascular protective effects of berberine have been reported in experimental studies of diverse vascular diseases. Berberine has shown promising vascular protection against atherosclerosis, cerebrovascular disease, hypertension, diabetes mellitus (DM), and intestinal vascular diseases. The pharmacological properties and molecular pathways of berberine are presented in Figure 2.

### Vasculature in Heart

Cardiovascular disease (CVD) is a major cause of morbidity and mortality worldwide (Huang et al., 2021). Studies indicate that the abnormal proliferation of vascular smooth muscle cells (VSMCs) is involved in the pathogenesis of CVD (Xiang et al., 2020; Zhang et al., 2021a). It has also been demonstrated that berberine (10, 30 and 100 μmol/L) could inhibit angiotensin IV-induced proliferation in cultured VSMCs by targeting the peroxisome proliferator-activated receptor α (PPAR-α)–nitric oxide (NO) signaling pathway (Qiu et al., 2017).

#### Myocardial Ischemia

Inflammation is one of the most observed cardiovascular conditions, and has a significant role in the progression of CVD (Golia et al., 2014; Lockshin et al., 2018). Aggravating inflammation may induce vascular remodeling after myocardial ischemia (MI), contributing to reduction of the ejection fraction and subsequent heart failure (Lu et al., 2015; Chong et al., 2021). It has been suggested that 50 mg/kg berberine may improve vascular inflammation and remodeling by inhibiting p38 mitogen-activated protein kinase (MAPK) activation, and activating transcription factor 2 phosphorylation (p-ATF-2) and matrix metalloprotease 2 (MMP-2) expression in rats (Li et al., 2015a). Protein hyperacetylation is associated with the development of MI (Treviño-Saldaña and García-Rivas, 2017; Aggarwal et al., 2020). Accumulating studies have demonstrated that silent information regulator 1 (SIRT-1) can regulate oxidative stress and inflammation to inhibit the development and progression of cardiac dysfunction in myocardial ischemia/reperfusion (MI/R) injury (Xue et al., 2020; Chang et al., 2021). Owing to its strong antioxidative and anti-inflammatory activities, oral administration of berberine (200 mg/kg) conferred cardioprotective effects in rats by improving post-MI/R cardiac function recovery and reducing infarct size after MI/R injury; the mechanism of action was found to be associated with the regulation of the SIRT-1 signaling pathway (Yu et al., 2016). As a selective barrier between tissue and blood, endothelial cells play a potential role in the control of inflammatory responses and homeostasis (Krüger-Genge et al., 2019). Endothelial cell dysfunction and/or injury can disrupt the integrity of the endothelial lining and subsequently lead to vascular disease, such as MI (Monteiro et al., 2019). Additionally, large experimental studies suggest that excessive inflammation can directly lead to endothelial cell apoptosis (Bravo-San Pedro et al., 2017; Henning et al., 2018). Lipopolysaccharide (LPS)-induced inflammation and apoptosis in human umbilical vein endothelial cells (HUVECs) were found to be inhibited by pretreatment with 5 μM berberine, mediated by inhibition of c-Jun N-terminal kinase (JNK) phosphorylation, and increased myeloid cell leukemia 1 (MCL-1) expression and superoxide dismutase (SOD) activity (Guo et al., 2016).

Angiogenesis, the formation of new blood vessels from preexisting ones, is indispensable for revascularization and cardiac remodeling following MI (Mathiyalagan et al., 2019; Chong et al., 2021; Tang et al., 2021). Ischemic heart disease is a leading cause of mortality and results from vascular cavity stenosis and occlusion (Garry et al., 2021). Rehabilitation of the myocardial ischemic region involves the activation of several stimulatory and inhibitory modulators of angiogenesis; the most notable of which are vascular endothelial growth factor (VEGF), fibroblast growth factor 2 (FGF-2), and thrombospondin 1 (TSP-1) (Detillieux et al., 2003; Frangogiannis et al., 2005; Martinez et al., 2018; Garikipati et al., 2019). Treatment with 10 mg/kg of berberine-rich extract (5 days a week by gavage) remarkably reduced heart infarct size, and increased the expression of angiogenesis-promoting factors in rats with MI/R injury, including VEGF, FGF-2 and TSP-1 (Banaei et al., 2020). In addition, microRNA plays a key role in many cardiac pathological processes, including MI (Parikh et al., 2020). Treatment with berberine in mice with MI injury was shown to lead to elevated miR-29b can activate the protein kinase B (Akt) signaling pathway, thus promoting angiogenesis and cell proliferation and migration to improve heart function (Zhu et al., 2017). A study in zebrafish embryos revealed that the level of VEGF-aa mRNA was up-regulated by berberine, which interfered with the angiogenic process, promoting bradycardia and reducing the cardiac output, atrial shortening fraction percentage, and atrial stroke volume (Martini et al., 2020). A complex hemodynamic pathological phenomenon exists in ischemia and reperfusion injury that can engage the metabolic and inflammatory machinery in the development of various disorders, including heart failure (Raza et al., 2020). Interestingly, intragastric administration of 100 mg/kg berberine daily for 14 days attenuated ischemia–reperfusion injury via hemodynamic improvements and inhibition of AMPK activity in both non-ischemic and ischemic areas of rat heart tissue (Chang et al., 2012).

**FIGURE 3:**
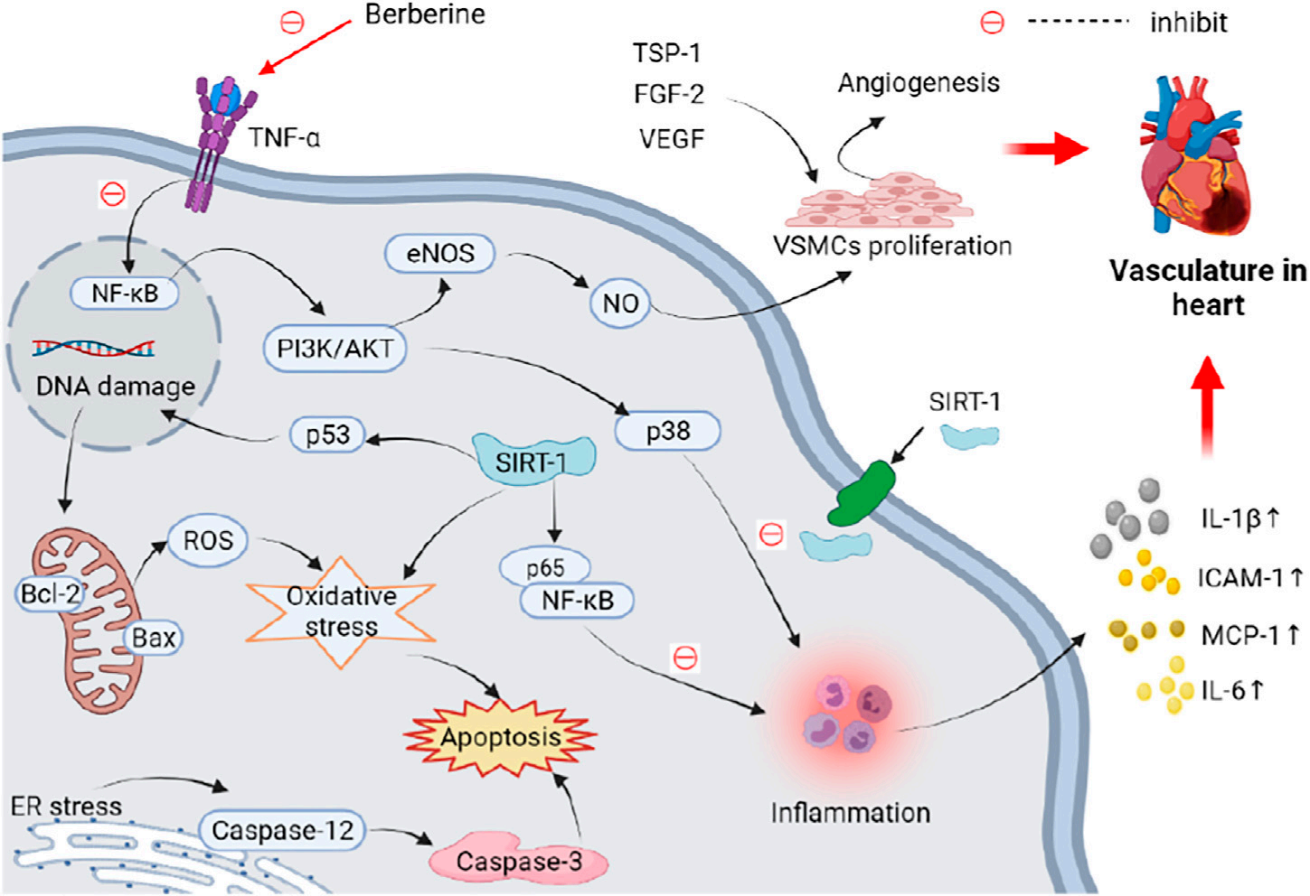
Schematic representation of the possible mechanism of anti-vasculature in heart activity of berberine. Elevated miR-29b can activate the Akt signaling pathway, thus promoting angiogenesis and cell proliferation and migration to improve vasculature in heart. Increased inflammation, oxidative stress, and ER stress can induce apoptosis of vascular endothelial cells. These adverse effects on vasculature in heart can be reversed by berberine. Abbreviations: Akt, protein kinase B; Bax, Bcl-2 associated X protein; Bcl-2, B-cell lymphoma 2; eNOS, endothelial nitric oxide synthase; ER, endoplasmic reticulum; FGF-2, fibroblast growth factor 2; ICAM-1, intercellular adhesion molecule 1; IL-1β, interleukin 1β; MAPK, mitogen-activated protein kinase; MCP-1, monocyte chemoattractant protein 1; NF-κB, nuclear factor κB; PI3K, phosphoinositide 3-kinase; ROS, reactive oxygen species; SIRT-1, silent information regulator 1; TNF-α, tumor necrosis factor α; TSP-1, thrombospondin 1; VEGF, vascular endothelial growth factor; VSMCs, vascular smooth muscle cells.

Recent evidence has confirmed that endoplasmic reticulum (ER) stress is correlated with the development and progression of various heart diseases including cardiac hypertrophy, ischemic heart diseases, and heart failure (Wang et al., 2018). Prolonged ER stress, however, can become a leading cause of vascular endothelial cell dysfunction and apoptosis in CVD (Chen et al., 2020; Fatima et al., 2021). Oral administration of 200 mg/kg berberine daily for 2 weeks was reported to protect the heart from MI/R injury in rats by activating the janus kinase 2 (JAK-2)/signal transducer and activator of transcription 3 (STAT-3) signaling pathway, as well as by attenuating ER stress-induced apoptosis (Zhao et al., 2016). Alternatively, apoptosis and inflammation are correlated with anoxia-reoxygenation injury in CVD, which typically occurs during MI (Huang et al., 2018; Gan et al., 2020). The decreased inflammatory cytokines and myocardial cell apoptosis resulting from berberine administration may alleviate anoxia-reoxygenation injury by downregulating p38 MAPK-mediated nuclear factor κB (NF-κB) signaling pathway (Zhao et al., 2019c). In conclusion, given its robust anti-inflammatory, antioxidative stress, antiapoptotic, and anti-ER stress effects, berberine may effectively improve CDV and MI by inhibiting the MAPK, AMPK and NF-κB pathways and activating PPARα-NO, VEGF, and JAK-2/STAT-3 pathways (Figure 3 and Table 1).

**TABLE 1:** Pharmacological properties of berberine in vasculature in heart.

Subjects	Model	Doses/Duration	Effects/Mechanisms	References
*In vivo*				
Wistar (male, 8–12 weeks, 240 ± 20 g)	MI/R	10 mg/kg, i.g., for 8 weeks	Infarct size↓, cardiac output↑, EF↑, and FS↑; mRNA: TSP-1↓, VEGF↑, and FGF-2↑; protein: CK-MB↓ and caspase-3↓	Banaei et al. (2020)
Wistar (male, 6 weeks, 240 ± 20 g)	Obese-diet and high sugar drinking for 16 weeks	50 mg/kg, i.g., for 8 weeks	Body weight↓, cholesterol↓, glucose↓, insulin↓, and HOMA-IR↓; serum: TNF-α↓, and IL-6↓; mRNA: TNF-α↓, IL-6↓, ICAM-1↓, and VCAM-1↓; protein: ICAM-1↓, VCAM-1↓, MMP-2↓, p-p38↓, and p-ATF-2↓	Li et al. (2015a)
Wistar (male, 250–280 g)	MI/R	100 mg/kg, i.g., for 2 weeks	LVEDP↓, +dP/dt↑, -dP/dt↑, and LVDP↑; preotein: p-AMPK/AMPK↓, AMP/ATP↓, and ADP/ATP↓	Chang et al. (2012)
SD (male, 200–250 g)	MI/R	100 mg/kg, i.g. for 2 weeks	Apoptotic index↓, infarct size↓, LVFS↑, and LVEF↑; serum: LDH↓, CK activity↓, MDA↓, and SOD↑; protein: caspase-3↓, Bcl-2↑, Bax↓, p-pERK/pERK↓, p-elF-2α/elF-2α↓, ATF-4↓, CHOP↓, p-JAK-2/JAK-2↑, and p-STAT-3/STAT-3↑	Zhao et al. (2016)
C57BL/6 (male, 8 weeks, 28–32 g)	Anoxia-reoxygenation injury	10 mg/kg, i.g., for 30 days	Apoptosis↓, BW↑, blood pressure↑, and heart rate↑; serum: blood lipid↓, IL-6↓, TNF-α↓, IL-10↓, and IL-17A↓; mRNA and protein: IL-6↓, TNF-α↓, IL-10↓, IL-17A↓, Bcl-2↑, and Bcl-xl↑	Zhao et al. (2019c)
SD (male, 7–9 weeks, 250–300 g)	MI/R	Pretreatment with 200 mg/kg, i.g., for 2 weeks	Infarct size↓, LVEF↑, and LVFS↑; serum: LDH↓, TNF-α↓, and CK activity↓; protein: superoxide generation↓, gp91^phox^↓, MDA↓, IL-6↓, TNF-α↓, MPO↓, caspase-3↓, Bax↓, SOD↑, SIRT-1↑, and Bcl-2↑	Yu et al. (2016)
C57BL/6 (male, 8 weeks, 20–25 g)	MI	100 mg/kg, through regular diet (1%, w/w), for 6 weeks	Infarct size↓ and microvascular density (CD-31↑, α-SMA↑); mRNA: miR-29b↑; protein: p-Akt↑	Zhu et al. (2017)
***In vitro***				
Primary VSMCs	Ang IV, 0.1 nM	30 μM	Cell proliferation↓, NOS↑, and NO↑; mRNA: PPAR-α↑ and eNOS↑; protein: PPAR-α↑	Qiu et al. (2017)
Zebrafish (48, 72, 96 and 120 hpf)	NA	100 mg/L, for 24, 48, and 96 h	mRNA: VEGF-aa↑ and PHD-3↑	Martini et al. (2020)
H9C2 embryonic rat myocardium-derived cells	Ischemic buffer for 2 h, then to normal culture medium for 4 h	50 μM	Apoptosis index↓; protein: Bax↓, p-pERK/pERK↓, p-elF-2α/elF-2α↓, ATF-4↓, CHOP↓, p-JAK-2/JAK-2↑, p-STAT-3/STAT-3↑, caspase-3↓, and Bcl-2↑	Zhao et al. (2016)
VSMCs	NA	NA	Cell apoptosis↓; mRNA and protein: p38↓, NF-κB↓, Bcl-2↑, and Bcl-xl↑	Zhao et al. (2019c)
H9C2 embryonic rat myocardium-derived cells	Ischemic buffer for 2 h, then to normal culture medium for 4 h	50 μM, for 8 h	Cell apoptosis↓ and viability↓; protein: superoxide generation↓, gp91^phox^↓, IL-6↓, TNF-α↓, caspase-3↓, Bax↓, SIRT-1↑, and Bcl-2↑	Yu et al. (2016)
HUVECs	NA	10, 25, 50, 100, and 200 μM, for 24 h	Cell proliferation↑ and migrations↑; mRNA: miR-29b↑; protein: p-Akt↑	Zhu et al. (2017)
HUVECs	5 μg/ml LPS for 24 h	Pretreatment with 1.25, 2.5, or 5 μM, for 24 h	Cell viability↑ and apoptosis↑; protein: MDA↓, IL-6↓, TNF-α↓, p-JNK↓, SOD↑, and MCL-1↑; mRNA: PARP↓ and MCL-1↑	Guo et al. (2016)

(Increase, ↑; Decrease, ↓). Abbreviations: AAR, area at risk area; ADP, adenosine diphosphate; Akt, protein kinase B; AMP, adenosine monophosphate; AMPK, AMP-activated protein kinase; ATF-2, activating transcription factor 2; ATP, adenosine triphosphate; Bax, Bcl-2 associated X protein; Bcl-2, B-cell lymphoma 2; BW, body weight; CHOP, C/EBP homologous protein; CK, creatine kinase; CRP, c-reactive protein; EDV, end diastolic volume; EF, ejection fraction; eIF-2α, eukaryotic initiation factor 2α; eNOS, endothelial nitric oxide synthase; ERK, extracellular signal-regulated kinase; ESV, end systolic volume; FGF-2, fibroblast growth factor 2; FS, fractional shortening; HOMA-IR, homeostasis model assessment-estimated insulin resistance; HUVECs, human umbilical vein endothelial cells; ICAM-1, intercellular adhesion molecule 1; IL-6, interleukin 6; IS, infract size; JAK-2, janus kinase 2; JNK, c-Jun N-terminal kinase; LDH, lactate dehydrogenase; LPS, lipopolysaccharide; LVEDP, left ventricular end-diastolic pressure; LVEF, left ventricular ejection fraction; LVFS, left ventricular fractional shortening; MAPK, mitogen-activated protein kinase; MCL-1, myeloid cell leukemia 1; MDA, malondialdehyde; MI/R, myocardial infarction/reperfusion; MMP-2, matrix metalloproteinase 2; MPO, myeloperoxidase; NA, not available; NOS, nitric oxide synthase; Pak-1, p21-activated kinase 1; PARP, poly (ADP-ribose) polymerase; PHD-3, prolyl hydroxylase 3; PP2B, calcineurin; PPAR-α, peroxisome proliferator-activated receptor α; SIRT-1, silent information regulator 1; Smad-3, small mother against decapentaplegic 3; SOD, superoxide dismutase; STAT-3, signal transducer and activator of transcription 3; SV, stroke volume; TGF-β, transforming growth factor β; TNF-α, tumor necrosis factor α; TSP-1, thrombospondin 1; VCAM-1, vascular cell adhesion molecule 1; VEGF, vascular endothelial growth factor; VF, ventricular fibrillation; VSMCs, vascular smooth muscle cells; VT, ventricular tachycardia.

#### Atherosclerosis

Atherosclerosis is a leading cause of death worldwide, and is characterized by lipid deposition, chronic inflammatory injury, smooth muscle cell proliferation, and plaque formation (Insull, 2009; Wolf and Ley, 2019). The pathological process of atherosclerosis begins with endothelial damage, accompanied by abnormal migration of VSMCs, leading to vascular remodeling (Sitia et al., 2010; Liang et al., 2020). Administration of 25, 50 and 100 μM berberine may suppress the expression of MMP-2, MMP-9 and urokinase-type plasminogen activator (u-PA) to significantly inhibit fetal bovine serum-induced human aortic smooth muscle cell (HASMC) migration, which may act to interrupt the activator protein 1 (AP-1) and NF-κB signaling pathways (Liu et al., 2014). Dysregulation of lipid metabolism is considered another major risk factor for atherosclerosis (Agrawal et al., 2018). An expert committee published the National Cholesterol Education Program in United States, emphasizing that low density lipoprotein (LDL) should be the primary target of cholesterol-lowering therapy in atherosclerosis (Tavares et al., 2021). In particular, clinical trials have demonstrated that lowering LDL levels can reduce the risk of atherosclerosis (Ference et al., 2017; Ji and Lee, 2021). Notably, the increased lipid in the serum and liver was reduced with the administration of berberine, which improved intima-media thickening, restored aortic endothelium-dependent vasodilatation, and alleviated atherosclerotic lesions in APOE^(−/−)^ mice fed a western-type diet for 12 weeks (Tan et al., 2020). Similarly, berberine ameliorated high-fat diet (HFD)-induced hyperlipidemia and lipid accumulation in liver and adipose tissue, alleviated endothelial lesions and reduced the expression of inflammatory cytokines in the plasma of APOE^(−/−)^ mice; it also reduced cholesteryl ester gathering in the aortic arch, resulting in ameliorated arterial plaque build-up via altered AMPK and NF-κB gene expression, and interrupted crosstalk between adipocytes and macrophages (Ma et al., 2020).

Tumor necrosis factor α (TNF-α) is a major proinflammatory factor in the development of vascular inflammation (Jang et al., 2017). Aberrant inflammatory responses may result in the ablation of macrophages, aggravation of vascular endothelial injury, and abnormal tissue proliferation in atherosclerosis (Hui et al., 2010; Brown et al., 2017). *In vitro* studies have indicated that TNF-α-induced inflammation, which causes excessive expression of intercellular adhesion molecule-1 (ICAM-1) and monocyte chemoattractant protein 1 (MCP-1) could be decreased by berberine in human aortic endothelial cells; this may be associated with inhibition of the NF-κB and AMPK pathways (Liu et al., 2015b). Oxidized LDL (ox-LDL) can act as an antigen to activate the immune inflammatory response, increasing the infiltration of inflammatory cells and the secretion of inflammatory factors in atherosclerosis (Lundberg et al., 2021). Ox-LDL-induced HUVEC proliferation and inflammatory responses were reversed with berberine, which lowered the expression of proliferating cell nuclear antigen (PCNA), NF-кB, and lectin-like oxidized low-density lipoprotein receptor 1 (LOX-1), and inhibited the phosphoinositide 3-kinase (PI3K)/Akt, extracellular signal-regulated kinase 1/2 (ERK-1/2), and p38 MAPK pathways (Xu et al., 2017). Ox-LDL has also been shown to injure endothelial cells directly, and contributes to endothelial dysfunction via overexpression of LOX-1, which induces a further rise in intracellular reactive oxygen species (ROS) (Kattoor et al., 2019; Akhmedov et al., 2021). Orally administrated at 156 mg/kg, berberine improved endothelial dysfunction by reducing aortic ROS generation and the release of inflammatory cytokines in the serum of a mouse model of atherosclerosis (Tan et al., 2020). Additionally, platelet–endothelial cell interactions potentiated by oxidative stress are thought to contribute to early atherosclerosis (Brown et al., 2021). Existing inflammatory and oxidative suppression of berberine prevented the development of the atherosclerotic plaque area by inhibiting translocation of NF-κB to the nucleus (Feng et al., 2017). Uncoupling protein 2 (UCP-2) is an inner mitochondrial membrane protein that belongs to the UCP family and plays an important role in lowering mitochondrial membrane potential and dissipating metabolic energy, preventing the accumulation of oxidative stress (Pierelli et al., 2017). Amazingly, treatment with 1 mmol/L berberine in drinking water led to suppression of oxidative stress and vascular inflammation by stimulating AMPK-dependent UCP-2 expression in mice with atherosclerosis (Wang et al., 2011). Moreover, recent studies found that the gut microbiota played a crucial role in atherosclerosis (Jonsson and Bäckhed, 2017; Verhaar et al., 2020). Another study emphasized that the modulation of gut microbiota, specifically the abundance of the *Akkermansia* bacterial genus, contributed to the antiatherosclerotic and metabolic protective effects of berberine by suppressing intestinal inflammation and promoting intestinal epithelial barrier integrity (Zhu et al., 2018).

**FIGURE 4:**
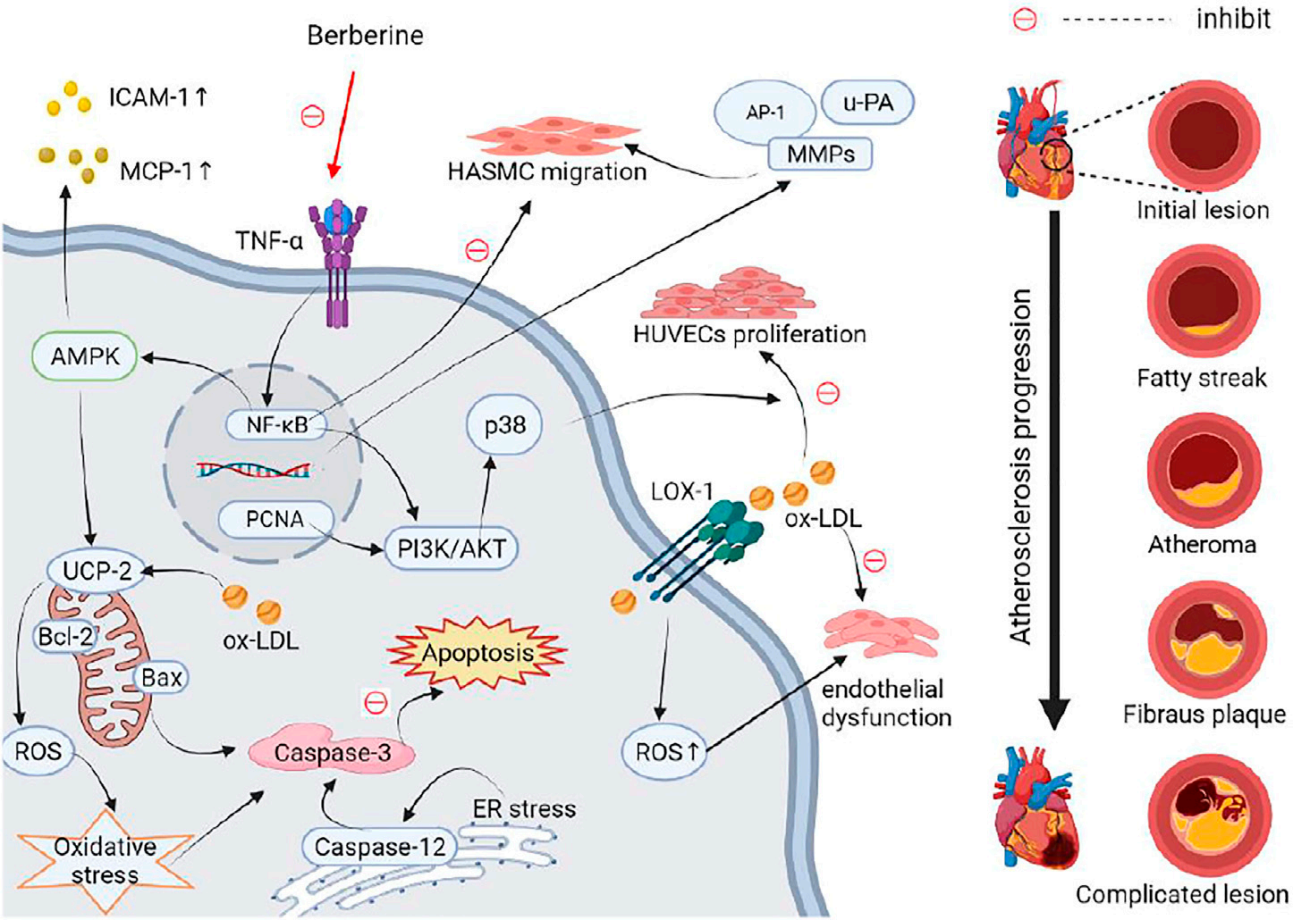
Schematic representation of the possible mechanism of anti-atherosclerosis activity of berberine. The dysregulation of lipid metabolism, vascular endothelial damage can induce abnormal migration of VSMCs, leading to vascular remodeling in atherosclerosis. Increased ox-LDL and LOX-1 cause vascular endothelial dysfunction. Inflammation leads to excessive expression of ICAM-1 and MCP-1, and aggravation of vascular endothelial proliferation and migration via up-regulating NF-κB and AMPK pathways. Inflammation, oxidative stress, and ER stress aggravate vascular endothelial cell apoptosis in atherosclerosis. These adverse effects on atherosclerosis can be reversed by berberine. Abbreviations: Akt, protein kinase B; AMPK, AMP-activated protein kinase; AP-1, activator protein 1; Bax, Bcl-2 associated X protein; Bcl-2, B-cell lymphoma 2; ER, endoplasmic reticulum; HASMC, human aortic smooth muscle cell; ICAM-1, intercellular adhesion molecule 1; LOX-1, low-density lipoprotein receptor 1; MAPK, mitogen-activated protein kinase; MCP-1, monocyte chemoattractant protein 1; MMPs, matrix metalloproteases; NF-κB, nuclear factor κB; Ox-LDL, oxidized low density lipoprotein; PCNA, proliferating cell nuclear antigen; PI3K, phosphatidylinositol 3-kinase; ROS, reactive oxygen species; TNF-α, tumor necrosis factor α; UCP-2, uncoupling protein 2; u-PA, urokinase-type plasminogen activator.

Prolonged activation of the ER stress pathway can lead to aggravated oxidative stress and endothelial cell apoptosis (Tabas, 2010; Linton et al., 2016). Kawasaki disease (KD) is an acute febrile illness characterized by systemic vasculitis, especially in coronary arteries (de Ferranti et al., 2018). Berberine exerted its protective effects on KD-induced apoptosis of human coronary artery endothelial cells by inhibiting oxidative and ER stress (Xu et al., 2020). Endothelial cell apoptosis induced by ER stress is closely linked with plaque progression, which can contribute to unstable atherosclerotic plaques, perhaps in response to thrombosis in atherosclerosis (Yang et al., 2021). Homocysteine increases damage to vascular endothelial cells, thereby reducing vasodilation factors released by endothelial cells and impairing vasodilation in the endothelium, resulting in vascular endothelial apoptosis and inducing oxidation (Salvio et al., 2021). Nevertheless, an *in vivo* experiment has shown that berberine increased the stability of atherosclerotic plaques and mitigated detrimental effects of vascular endothelial cell activity experimentally induced by 50 mg/kg homocysteine thiolactone, and similar results are found in vitro study induced by 1 mM homocysteine thiolactone (Li et al., 2016a). Collectively, the studies presented here (Table 2) indicate that berberine may improve vascular endothelial damage, abnormal lipid metabolism, chronic inflammation, plaque formation, and cell apoptosis, thereby alleviating atherosclerosis (Figure 4).

**TABLE 2:** Pharmacological properties of berberine in atherosclerosis.

Subjects	Model	Doses/Duration	Effects/Mechanisms	References
*In vivo*				
ApoE^-/-^ mice (male, 6–8 weeks)	Atherosclerosis	78 and 156 mg/kg, i.g., for 12 weeks	Liver index↓; serum: FFA↓, TG↓, TC↓, ox-LDL↓, MDA↓, IL-6↓, ET-1↓, TUNEL-positive cells↓, and e-NOS↑; protein: GPD-2↓, PON-1↑, and APOA-1↑	Tan et al. (2020)
ApoE^-/-^ mice (male, 6–8 weeks, 20–22 g)	Atherosclerosis	100 mg/kg, i.g., for 5 months	Endothelial injury↓, atherosclerotic lesions↓, adipose sise↓, and macrophages infiltration↓; plasma: TG↓, cholesterol↓, LDL-C↓, and cholesteryl↑; serum: IL-6↓, IL-1β↓, IFN-γ↓, TNF-α↓, and MCP↓; liver and adipose: IL-6↓, IL-1β↓, TNF-α↓, NF-κB↓, and p-AMPK↓; protein: ICAM-1↓, VCAM-1↓, and MMP↓	Ma et al. (2020)
ApoE^-/-^ mice (6 weeks)	Atherosclerosis	150 mg/kg, i.g., for 12 weeks	plasma: T-AOC↑ and CAT↑; serum: TC↓, TG↓, LDL-C↓, IL-1β↓, and TNF-α↓; mRNA and protein: NF-κB p65↓, iNOS↓, ICAM-1↓, and IL-6↓	Feng et al. (2017)
ApoE^-/-^ mice (5 weeks)	Atherosclerosis	1 mmol/L, in drinking water, for 8 weeks	Atherosclerotic lesions↓; protein: ICAM-1↓, VCAM-1↓, 4-HNE↓, MDA↓, 3-NT↓, UCP-2↑, p-AMPK↑, and p-ACC↑	Wang et al. (2011)
ApoE^-/-^ mice (male, 5 weeks)	Atherosclerosis	0.5 g/L, in drinking water, for 14 weeks	Atherosclerotic lesions↓ and plaque area↓; serum: TC↓, TG↓, IL-1β↓, and TNF-α↓; intestine: IL-1β↓, TNF-α↓, ZO-1↑, and occludin↑; mRNA and protein: VCAM-1↓ and MMP-2↓; gut microbiota: Akkermansia↑	Zhu et al. (2018)
ApoE^-/-^ mice (male, 8–12 weeks)	Atherosclerosis	1 g/kg, i.g., for 8 weeks	Carotid atherosclerotic plaque stability↑ and vascular relaxation↑; serum: MDA↓ and NO↑; protein: MDA↓ and SOD↑	Li et al. (2016a)
*In vitro*				
HASMC	NA	100 μM, for 24 h	Cell migration↓; protein: c-Fos↓, AP-1↓, and NF-κB↓; mRNA and protein: MMP-2↓, MMP-9↓, and u-PA↓	Liu et al. (2014)
HAECs	10 ng/ml TNF-α for 30 min	5, 10, and 25 μM, for 1 h	Protein: NF-κB p65↓, p-AMPK/AMPK↓ and p-ACC/ACC↑; mRNA and protein: ICAM-1↓ and MCP-1↓	Liu et al. (2015b)
HUVECs	50 μg/ml ox-LDL for 24 h	1, 5, 10, 25, and 50 μg/ml, for 1 h	Cell proliferation↓; mRNA: PCNA↓, NF-κB↓, LOX-1↓ and PI3K↓; protein: PCNA↓, LOX-1↓, NF-кB↓, p-Akt/Akt↓, p-ERK/ERK↓, and p-p38/p38↓	Xu et al. (2017)
HUVECs	NA	10 μM, for 2 h	mRNA and protein: UCP-2↑	Wang et al. (2011)
HCAECs	Serum from KD patients or healthy volunteers for 24 h	20 μM, for 24 h	Cell apoptosis↓; protein: ROS↓, THBD↓, vWF↓, EDN-1↓, ATF-4↓, p-eIF-2α↓, p-PERK↓, and XBP-1↓	Xu et al. (2020)
HUVECs	1 mM homocysteine thiolactone for 24 h	10, 50, and 100 μM, for 1 h	Cell viabilities↑ and ROS↓	Li et al. (2016a)

(Increase, ↑; Decrease, ↓). Abbreviations: 3-NT, 3-nitrotyrosine; 4-HNE, 4-hydroxynoneal; ACC, acetyl-CoA carboxylase; Akt, protein kinase B; AMPK, AMP-activated protein kinase; APOA-1, apolipoprotein A1; CAT, catalase; CPT-1α, carnitine palmitoyl transferase 1α; eIF-2α, eukaryotic initiation factor 2α; ERK, extracellular signal-regulated kinase; ET-1, endothelin 1; FABP-4, fatty acid binding protein 4; FFA, free fatty acids; GPD-2, glycerol-3-phospate dehydrogenase 2; GSH, glutathione; HAECs, human aortic endothelial cells; HASMC, human aortic smooth muscle cell; HFD, high-fat diet; HUVECs, human umbilical vein endothelial cells; ICAM-1, intercellular adhesion molecule 1; IFN-γ, interferon γ; IL-6, interleukin 6; iNOS, inducible nitric oxide synthase; KD, Kawasaki disease; LDL-c, low density lipoprotein cholesterol; LOX-1, low-density lipoprotein receptor 1; LPL, lipoprotein lipase; MCP, monocyte chemoattractant protein; MDA, malondialdehyde; MMP, matrix metalloprotease; NA, not available; NF-κB, nuclear factor κB; Ox-LDL, oxidized low density lipoprotein; PCNA, proliferating cell nuclear antigen; PI3K, phosphoinositide 3-kinase; PON-1, paraoxonase 1; PPAR-γ, peroxisome proliferator-activated receptor γ; SOD, superoxide dismutase; T-AOC, total antioxidant capacity; TC, total cholesterol; TG, triglyceride; THBD, bovine thrombin regulatory protein; TNF-α, tumor necrosis factor α; UCP-2, uncoupling protein 2; u-PA, urokinase-type plasminogen activator; VCAM-1, vascular cell adhesion molecule 1; vWF, von willebrand factor; XBP-1, X-box binding protein 1; ZO-1, zona occluden 1.

### Cerebrovascular Diseases

#### Alzheimer’s Disease

Alzheimer’s disease (AD) is a complex, aging-related, neurodegenerative disease, and the pathology process mainly involves β-amyloid (Aβ) overproduction and accumulation, tau hyperphosphorylation, and neuronal loss (Lian et al., 2016; Spangenberg et al., 2016; Gao et al., 2018). Administration of 0.5 μM berberine significantly suppressed Aβ-induced production of interleukin 6 (IL-6) and MCP-1 by inhibiting the activation of NF-κB and blocking the PI3K/Akt and MAPK pathways (Jia et al., 2012). Accumulated Aβ can result from abnormal processing of amyloid precursor protein (APP) in AD (O'Brien and Wong, 2011). APP C-terminal fragment levels and APP and tau hyperphosphorylation were decreased with 100 mg/kg berberine, administered by oral gavage for 4 months, via the Akt/glycogen synthase kinase 3 (GSK-3) pathway in rats with AD (Durairajan et al., 2012). Similarly, berberine modulates Aβ generation through activation of the AMPK pathway in N2a mouse neuroblastoma cells (Zhang et al., 2017). Moreover, elevated Aβ can mediate synaptic loss and dysfunction, another pathological hallmark of AD, by targeting mitochondria (Hong et al., 2016). Pretreatment with 1 μM berberine alleviated axonal mitochondrial abnormalities by preserving the mitochondrial membrane potential and preventing decreases in ATP, increasing axonal mitochondrial density and length, and improving mitochondrial motility and trafficking in 0.5 μM Aβ cultured hippocampal mouse neurons (Zhao et al., 2019a). Additionally, neuronal loss and cerebral blood flow contributed to dysfunction in memory and reward systems in AD (Dai et al., 2009; Nobili et al., 2017). Studies found that drinking water containing 100 mg/L berberine exerted a strong neuroprotective effect, improving cognitive deficits, inhibiting the apoptosis of neurons, and promoting the formation of micro-vessels by restoring cerebral blood flow and reducing Aβ accumulation in an APP/tau/PS-1 mouse model of AD (Ye et al., 2021). As shown in Table 3, the potential effects of berberine observed in the study suggest that it may effectively prevent AD by reducing accumulated Aβ. Few studies have focused on the improvement of tau hyperphosphorylation and neuronal loss in AD, which should thus be a focus of future research.

**TABLE 3:** Pharmacological properties of berberine in the treatment of cerebrovascular disease.

Subjects	Model	Doses/Duration	Effects/Mechanisms	References
*In vivo*				
TgCRND8 mice	AD	25 and 100 mg/kg, i.g., for 16 weeks	Learning and memory↑, plaque load↓, microgliosis, and astrogliosis↓; protein: Aβ↓, p-APP↓, PHF-1↓, AT-8↓, AT-180↓, GSK-3↓, tau-1↑, and p-Akt/Akt↑	Durairajan et al. (2012)
3 x Tg AD mice (male, 6 months)	AD	100 mg/kg, in drinking water, for 16 weeks	Cerebral blood flow↑ and cognitive impairments↓; hippocampus protein: Aβ↓, GFAP↓, caspase-3↓, NeuN↑, N-cadherin↑, VEGF↑, Ang-1↑, and CD-31↑	Ye et al. (2021)
*In vitro*				
Primary microglial and BV2 cells	20 mm Aβ for 48 h	1, 2.5, and 5 μM, for 30 min	Protein: NF-κB p65↓, p-IκB-α↓, p-ERK/ERK↓, p38/p38↓, and p-Akt/Akt↓, mRNA and protein: IL-6↓, MCP-1↓, iNOS↓, and COX-2↓	Jia et al. (2012)
N2a/APP695sw, N2a cellsN2a cells and primary cortical neurons	NA	1 and 3 μM, for 24 h	Protein: Aβ↓ and p-AMPK↑; mRNA and protein: BACE-1↓	Zhang et al. (2017)
Primary hippocampal neuron cells	oligomeric Aβ1- 42 (0.5 μM) for 24 h	0.1, 0.3, and 1 μM for 1 h	Synaptic loss↓, axonal mitochondrial index↑, average lengths of axonal mitochondria↑, distribution of axonal mitochondrial lengths↑; protein: MDA↓, ATP↑	Zhao et al. (2019a)
HAECs	10 ng/ml TNF-α for 30 min	25 μM, for 1 h	Protein: NF-κB p65↓, p-AMPK/AMPK↓ and p-ACC/ACC↑; mRNA and protein: ICAM-1↓ and MCP-1↓	Liu et al. (2015b)

(Increase, ↑; Decrease, ↓). Abbreviations: 5-HT, 5-hydroxytryptamine; ACC, acetyl-CoA carboxylase; AD, Alzheimer’s disease; Akt, protein kinase B; AMPK, AMP-activated protein kinase; APP, amyloid precursor protein; ATF-2, activating transcription factor 2; ATP, adenosine triphosphate; Aβ, β amyloid; BACE-1, β-site APP cleaving enzyme 1; Bax, Bcl-2 associated X protein; CBF, cerebral blood flow; CD-31, platelet endothelial cell adhesion molecule 1; COX-2, cyclo-oxygenase 2; CTFs, c-terminal fragments; Cyto-c, cytochrome c; ERK, extracellular signal-regulated kinase; GFAP, glial fibrillary acidic protein; GSH, glutathione; GSK-3, glycogen synthase kinase 3; ICAM-1, intercellular adhesion molecule 1; IL-1β, interleukin 1β; iNOS, inducible nitric oxide synthase; MAO, monoamine oxidase; MCP-1, monocyte chemoattractant protein 1; MDA, malondialdehyde; NA, not available; NF-κB, nuclear factor κB; PHF-1, paired helical filament; SOD, superoxide dismutase; TNF-α, tumor necrosis factor α; VD, vascular dementia; VEGF, vascular endothelial growth factor.

#### Vascular Dementia

Vascular dementia (VD) is the second most common form of dementia and is caused by vascular pathologies causing brain injury (Poh et al., 2021). In a rat model of VD induced by cerebral ischemia–reperfusion injury, increased angiogenesis was observed with berberine chloride (50 mg/kg) treatment, which may be due to the activation of hypoxia-inducible factor 1α (HIF-1α)/VEGF signal pathway (Liu et al., 2018). In general, few studies have focused on the relationship between berberine and vascular in VD. According to the development of new biological in recent years, imaging technology is conducive to strengthen the study of berberine and cerebrovascular diseases. Second near-infrared II (NIR-II) imaging, a kind of biomedical imaging technology with characteristics of high sensitivity, high resolution, and real-time imaging, can visualize the vasculature in the brain (Guo et al., 2019a). Therefore, we can directly utilize the NIR-II to observe the improvement of the vascular in the brain by treatment with berberine.

### Hypertension

An epidemiological investigation showed that the incidence of hypertension increases with age across all countries, regions, or ethnicities, and is generally higher in industrialized countries than developing countries (Zhou et al., 2021). Hypertension is more common in the elderly, and mostly manifests as simple systolic hypertension; however, it is increasing in younger age groups (James et al., 2014; Zhang and Moran, 2017). Among the many mechanisms underlying arterial stiffness, endothelial dysfunction is believed to be a critical determinant for its onset and progression (Kostov and Halacheva, 2018; Safar, 2018). Owing to arterial stiffness, elevated arterial blood pressure can contribute to both extracellular matrix (ECM) deposition and remodeling or enhanced contractility or stiffness of VSMCs (Bertorello et al., 2015). *In vitro*, berberine (at concentrations of 1.25, 2.5, and 5 μM) considerably inhibited aortic endothelial cells in spontaneous hypertensive rats (SHRs) by suppressing cell proliferation, apoptosis, and down-regulating the expression of Toll-like receptor 4 (TLR-4), myeloid differentiation protein 88 (MYD-88), NF-κB, IL-6, and TNF-α (Wang and Ding, 2015). Numerous studies have verified that vascular endothelial cell dysfunction and lncRNA activity may together be associated with hypertension (Lorenzen and Thum, 2016; Konukoglu and Uzun, 2017). The levels of five lncRNAs were found to be modulated by the administration of 100 mg/kg berberine in mice with hypertention, which may preserve vascular endothelial cell function (Tan et al., 2021). Endothelial microparticles (EMPs) are extracellular vesicles that are shed by the endothelium as a result of activation, injury, or apoptosis of endothelial cells, and are considered important biomarkers of the status of endothelial cells and vascular function (Sun et al., 2016). Endothelial progenitor cells (EPCs) mobilized from bone marrow can migrate to the peripheral blood and differentiate into mature endothelial cells, contributing to endothelial recovery (Pyšná et al., 2019). One study found that EPC number and activity was significantly reduced among people with hypertension when compared to healthy individuals, and low circulating levels of EPCs may contribute to endothelial dysfunction (Waclawovsky et al., 2021). Abnormal circulating EMPs and EPCs in SHRs were ameliorated by berberine treatment associated with endothelial dysfunction and arterial stiffness in SHRs (Zhang et al., 2020). Moreover, the production of NO caused by oxidative stress, aging, and spontaneous hypertension increases endothelium-dependent contractions (EDCs), contributing to blunted endothelium-dependent vasodilation (Vanhoutte et al., 2005; Vanhoutte et al., 2017). Continuous ER stress can exert detrimental effects through a maladaptive, unfolded protein response, resulting in cellular defects and disturbed vascular function (Ren et al., 2021). However, incubation with 1 μM berberine has been shown to reduce EDCs by activating the AMPK pathway, thus inhibiting ER stress and ROS generation, leading to cyclo-oxygenase 2 (COX-2) downregulation in SHR carotid arteries (Liu et al., 2015).

Ca^2+^ signals regulate vascular function in endothelial cells; Ca^2+^ release from the ER and/or Ca^2+^ influx through ion channels at the endothelial cell membrane results in endothelium-dependent vasodilation and diminished vascular resistance (Cook et al., 2012; Ottolini et al., 2019; Wang et al., 2021). Notably, endothelial transient receptor potential vanilloid 4 (TRPV-4) channels are associated with hypertension, as they regulate Ca^2+^ concentrations (Ottolini et al., 2020). Long-term administration of berberine has been shown to directly induce vasorelaxation, decreasing blood pressure and vascular stiffness, by suppressing the activity of TRPV-4 channels (Wang et al., 2015). Additionally, mechanical stretching forces increase the proliferation and apoptosis of VSMCs by activating the protein disulfide isomerase (PDI) redox system. Berberine has been shown to inhibit the PDI ER system and the MAPK pathway, thereby attenuating the simultaneous increases in VSMC proliferation and apoptosis observed in response to mechanical stretching during hypertension (Wang et al., 2020). Collectively, these results demonstrate that berberine may effectively ameliorate endothelial dysfunction, arterial stiffness, and vascular remodeling, resulting in reduced hypertension, and which the mechanism may be related to the inhibition of ER stress and activation of the AMPK pathway. An increasing number of studies on hypertension have focused on the Ca^2+^ and TRPV-4 pathways in recent years (Table 4).

**TABLE 4:** Pharmacological properties of berberine in the treatment of hypertension and PAH.

Subjects	Model	Doses/Duration	Effects/Mechanisms	References
*In vivo*				
C57BL/6 J (male, 8–10 weeks)	Hypertension	100 mg/kg, in drinking water, for 2 weeks	Blood pressure↓, aortic endothelial dysfunction↓, cytokine-cytokine receptor interaction↓, PPAR↓, vascular smooth muscle contraction↑ and ECM-receptor↑; DE-lncRNAs: AK041185↓, AK044823↓, AK076651↓, BY077582↓, ENSMUST00000119528↓, ENSMUST00000161399↓, ENSMUST00000155185↓, NR_028,422↓, ENSMUST00000144849↑, ENSMUST00000147654↑, uc.335+↑, ENSMUST00000155383↑, ENSMUST00000123078↑, and TCONS_00029108↑; DE-mRNAs: Nppa↓, Chrm2↓, Cdh1↓, Pde4b↑, Itga8↑, and Hhip↑	Tan et al. (2021)
SHR (male, 4 weeks)	Hypertension	50 mg/kg, i.g. for 4 weeks	Blood pressure↓, CD-31+/CD-42−MPs↑, CFUs↑, EPCs↑, aPWV↓, and aortic elastin fiber↑	Zhang et al. (2020)
SHR (male, 32–40 weeks)	Hypertension	1 μM, for 12 h	EDCs↓, ER stress↓ and ROS↓; protein: p-eIF-2a↓, ATF-3/6↓, XBP-1↓, and COX-2↓	Liu et al. (2015a)
C57B16 (male, 8–12 weeks, 20–25 g)	150 mg/kg deoxycorticosterone acetate	100 mg/kg, in nomal diet, for 35 days	Blood pressure↓ and vessel relaxation↑; protein: TRPV-4↓	Wang et al. (2015)
SD (male, 200–250 g)	SU5416 (20 mg/kg) on day 1 and then exposed to hypoxia	100 mg/kg, i.g., for 4 weeks	PAH↓, ventricle hypertrophy↓, RVAW↓, RVID↓, and vessel wall thickness↓; protein: Trx-1↓ and β-catenin↓	Wande et al. (2020)
C57/BL6 (male, 8 weeks, average 25 g)	Sugen 5461 (20 mg/kg) and exposed to 10% O_2_ for 4 weeks	100 mg/kg, i.g., for 4 weeks	RVSP↓, RVH↓, Src activity↓, pulmonary vascular remodeling↓, right ventricular chamber size↓, and muscularization↓; protein: p-Src↓ and HIF-1α↓	Liu et al. (2019)
SD (male, 6 weeks, 200–250 g)	Sugen 5416 (20 mg/kg) and exposed to 10% O_2_ for 4 weeks	100 mg/kg, i.g., for 4 weeks	RVSP↓, RVID↓, RVHI↓, and media fraction thickness↓; protein: p-Akt/Akt↓, p-ERK/ERK↓, p-p38/p38↓, and PP2A-c↓	Luo et al. (2018)
C57/BL6 (male, 6 weeks,18–21 g)	exposure to 10% O_2_ for 4 weeks	20 and 100 mg/kg, i.p., for 4 weeks	RVSP↓, RV/(LV + S)↓, medial wall thickness↓, and medial wall area↓; protein: TGF-β↓ and BMPR-2↑	Chen et al. (2019)
*In vitro*				
Aortic endothelial cells	Cell from SD rats with spontaneous hypertension	1.25, 2.5, and 5 μM, for 24 h	Cell apoptosis↓ and proliferation↑; protein: TNF-α↓, IL-6↓, TLR-4↓, Myd-88↓, and NF-κB↓	Wang and Ding, (2015)
HPASMCs	3% oxygen for 24 h	10 μM, for 24 h	Protein: Trx-1↓ and β-catenin↓	Wande et al. (2020)
PASMCs	3% oxygen for 48 h	10 μM	Pulmonary vascular remodeling↓, cell migration↓, and invasion↓; protein: p-Src↓ and HIF-1α↓	Liu et al. (2019)
PASMCs	Norepinephrine (10^−5^ M) and 5% oxygen for 24 h	12 h	Cell proliferation↓ and migration↓; protein: p-p38/p38↓, PP2A-c↓, p-PP2A/PP2A↓, p-Akt/Akt↓, and p-ERK/ERK↓	Luo et al. (2018)
PASMCs	3% oxygen for 48 h	20 and 100 μM, for 48 h	Cell proliferation↓, pulmonary vessel muscularization↓, PCNA-positive cell/total cell↓, and total vessel↓; protein: TGF-β↓, p-Smad-2/3↓, PCNA↓, PPAR-γ↑, p-Smad-1/5↑, and BMPR-2↑	Chen et al. (2019)

(Increase, ↑; Decrease, ↓). Abbreviations: Akt, protein kinase B; aPWV, aortic pulse wave velocity; ATF-3/6, activating transcription factor 3/6; BMPR-2, bone morphogenetic protein type 2; BP, blood pressure; CD-31, platelet endothelial cell adhesion molecule 1; CFUs, colony-forming units; COX-2, cyclo-oxygenase 2; ECM, extracellular matrix; EDCs, endothelium-dependent contractions; eIF-2α, eukaryotic initiation factor 2α; EPCs, endothelial progenitor cells; ER, endoplasmic reticulum; ERK, extracellular signal-regulated kinase; HIF-1α, hypoxia-inducible factor 1α; HPASMCS, human pulmonary artery smooth muscle cells; MyD-88, myeloid differentiation factor 88; PAH, pulmonary arterial hypertension; PASMCs, pulmonary artery smooth muscle cells; PCNA, proliferating cell nuclear antigen; PI3K, phosphoinositide 3-kinase; PP2Ac, protein phosphatase 2Ac; PPAR, peroxisome proliferator-activated receptor; ROS, reactive oxygen species; RVAW, right ventricle anterior wall; RVH, right ventricular hypertrophy; RVHI, right ventricle hypertrophy index; RVID, right ventricle internal dimension in diastole; RVSP, right ventricular systolic pressure; Smad-3, small mother against decapentaplegic 3; TGF-β, transforming growth factor β; TRPV-4, transient receptor potential vanilloid 4; Trx-1, Thioredoxin 1; XBP-1, X-box binding protein 1.

#### Pulmonary Arterial Hypertension

Pulmonary arterial hypertension (PAH) is closely associated with extensive vascular remodeling, especially pulmonary arterial medial hypertrophy and muscularization, due to aberrant proliferation of pulmonary artery smooth muscle cells (PASMCs) resulting from hypoxia (Bisserier et al., 2021; Sharifi Kia et al., 2021). Hemodynamic and pulmonary pathological data showed that chronic hypoxia notably elevated the median width of pulmonary arterioles (Choudhary et al., 2011; Khoramzadeh et al., 2019). However, berberine substantially decreased pulmonary vascular remodeling in mice with hypoxia-induced PAH by inhibiting Akt/mammalian target of rapamycin (mTOR)/HIF-1α and transforming growth factor β (TGF-β) pathways, and activating bone morphogenetic protein type 2 (BMPR-2) receptor (Chen et al., 2019; Liu et al., 2019). Wande et al. (2020) demonstrated substantial proliferative activities in hypoxia-induced human PASMCs, were possibly mediated by berberine (at concentrations of 10 μM) via inhibiting the thioredoxin (TRX-1) and β-catenin pathways. Activation of protein phosphatase 2A (PP2A) has been shown to induce apoptosis of PASMCs in people with PAH (Ferron et al., 2011). Berberine may prominently attenuate proliferation and migration of PASMCs induced by norepinephrine, alleviating PAH via PP2A pathway (Luo et al., 2018). These results together show that berberine significantly suppresses pulmonary vascular remodeling and proliferation of vascular endothelial cells in PAH; the antiproliferative and antiapoptotic effects may be mediated by inhibition of the Akt/mTOR/HIF-1α and PP2A pathways (Table 4).

### Diabetes Mellitus and its Complications

#### Diabetes Mellitus

The main cause of DM and associated complications is a low level or lack of pancreatic islet function, including insulin resistance, resulting in disordered glucose metabolism in the body (Pearson, 2019; Han et al., 2021b). Sustained hyperglycemia progresses to various diabetic microangiopathies, including diabetic cardiovascular disease, nephropathy, neuropathy and retinopathy (Reddy et al., 2015; Montero et al., 2019; Tang and Yiu, 2020). Chronic inflammation caused by hyperglycemia is a major feature of DM (Saltiel and Olefsky, 2017). Adipose tissue macrophages (ATMs), including M1 ATMs, can induce inflammatory responses by producing pro-inflammatory cytokines such as TNF-α and IL-6, thus contributing to the induction of insulin resistance in DM (Russo and Lumeng, 2018). Oral berberine (50 mg/kg/day) markedly improved insulin resistance, reduced the inflammation and JNK, IKK-β, and NF-κB p65 phosphorylation by inhibiting M1 macrophage activation in adipose tissue (Ye et al., 2016). Owing to increased aldose reductase (AR) and NADPH oxidase (NOX) activities, platelet hyperreactivity and apoptosis during DM accounts for the accumulation of ROS (Vara et al., 2019; Ajjan et al., 2021). It was demonstrated that 50 mM glucose induced platelet aggregation, apoptosis and superoxide production, which was neutralized by 25 and 50 μM berberine via inhibiting of AR, NOX and GSH reductase activities (Paul et al., 2019). Similarly, 25 μM berberine had protective effects against endothelial injury by attenuating the generation of ROS, cellular apoptosis, NF-κB activation, and expression of adhesion molecules, which were induced by high glucose levels; it also enhanced the endothelium-dependent vasodilatation through the activation of the AMPK pathway (Wang et al., 2009).

Advanced glycation end products (AGEs) are produced via the nonenzymatic glycation reaction and are considered major pathogenic factors that trigger vascular complications in diabetes (Azegami et al., 2021). Incubation with 5 or 10 μg/ml berberine significantly inhibited AGE formation in high-glucose-AGEs-induced micro-endothelial injuries (Hao et al., 2011). Diabetic peripheral neuropathy, characterized by perivascular neuropathy, is reported to be involved in vascular disorders associated with diabetes (Olesen et al., 2021). The mesenteric artery, a part of the splanchnic circulation system, is involved in the regulation of arterial pressure (van Dijk et al., 2020). The iliac artery is mainly responsible for blood supply to the lower extremities and pelvic organs (Maier et al., 2020). Both of these sites are predisposed to large vascular lesions in diabetes. Oral administration of 200 mg/kg berberine markedly enhanced the nitrergic neural activity in the superior mesenteric artery and diminished the adrenergic function in the iliac artery, resulting in vasodilatation in diabetic rats (Geng et al., 2016; Zhao et al., 2019b).

Intracellular Ca^2+^, which is tightly controlled by Ca^2+^ channels and transporters, is an important messenger in VSMC dedifferentiation (Nagel et al., 2006). Elevated concentrations of intracellular Ca^2+^ is a primary stimulus for smooth muscle contraction; it is reported that diabetic vascular dysfunction is tightly coupled to the impairment of intracellular Ca^2+^ processing in VSMCs (Searls et al., 2010; Yang et al., 2020). Impaired cerebral arterial vasodilation can be alleviated by berberine in a diabetic rat model via down-regulation of the intracellular Ca^2+^ processing of VSMCs (Ma et al., 2016). Hyperglycemia and hypertension are the two primary risk factors for vascular disease in diabetic patients (Cryer et al., 2016; Petrie et al., 2018). Increases in intracellular Ca^2+^ along with decreases in K^+^ can lower the membrane potential and enhance the vasoconstriction response and the proliferation of VSMCs, leading to hypertension (Seki et al., 2017; Sun et al., 2021). Chronic administration of 100 mg/kg berberine reduced blood pressure and improved vasodilation in diabetic rats by activating the Ca^2+^-activated K^+^ channel (Ma et al., 2017). Aberrant miR-133a expression in endothelial cells induces endothelial dysfunction and impaired endothelium-dependent vasodilation, aggravating the pathogenesis of cerebrovascular diseases (Li et al., 2016b). Administration of berberine (1.0 g/kg for 8 weeks) reduced miR-133a expression and impairments in learning and memory, increasing the vasodilation in the middle cerebral artery to improve VD associated with diabetes (Yin et al., 2019). Furthermore, existing evidence suggests that hyperglycemia and hypoxia can trigger cerebrovascular dysfunction (Lefferts et al., 2016; Jin et al., 2019). Berberine at a concentration of 30 μM counteracted the attenuating effects of hypoxic/high-glucose conditions on the proliferation and migration of rat brain microvascular endothelial cells, which was in part mediated by the SIRT-1/HIF-1α/VEGF pathway (Mi et al., 2019). According to these studies, berberine had a hypoglycemic effect through improving insulin resistance, and has robust anti-inflammatory, antiapoptotic and antiendothelial injury effects in DM. Berberine appears to improve endothelial dysfunction by regulating Ca^2+^ and K^+^ channels in DM (Table 5).

**TABLE 5:** The *in vivo* and *in vitro* mechanism of berberine in the treatment of DM and its complications.

Subjects	Model	Doses/Duration	Effects/Mechanisms	References
*In vivo*				
C57BL/6 (male, 4 w)	HFD containing 60% fat for 18 weeks	50 mg/kg, i.g., for 2 weeks	FBG↓ and F4/80+/CD11c+/CD206− cells↓; serum: insulin↓, TNF-α↓, IL-6↓, and MCP-1↓; adipose tissue: TNF-α↓, IL-6↓, MCP-1↓, p-JNK↓, p-IKK-β↓, and NF-κB p65↓	Ye et al. (2016)
SD (male, ∼190 g)	HFD for 4 w and STZ (45 mg/kg, once, ip.)	50, 100, and 200 mg/kg, i.g., for 8 weeks	FBG↓, body weight↑, augmented contractile responsiveness of middle cerebral artery↓, 5-HT↓, Ca_L_ channel current densities↓, and Ca^2+^↓; serum: insulin↑	Ma et al. (2016)
SD (male, ∼190 g)	HFD for 4 w and STZ (45 mg/kg, once, ip.)	50, 100, and 200 mg/kg, i.g., for 8 weeks	FBG↓, systolic and diastolic blood pressure↓, body weight↑, relaxation of middle cerebral artery↑, BK_Ca_ whole-cell current densities↑, and BK_Ca_ open probability↑; mRNA and protein: β1-subunit↑	Ma et al. (2017)
SD (male, 8–10 w, 180 ± 20 g)	50 mg/kg STZ for 5 consecutive days	1.0 g/kg, i.g., for 8 weeks	Short-term learning and memory↑, spatial memory↑, PCA blood flow↑, and relaxation of cerebral middle artery↑; serum: NO↑ and MDA↓; protein: BH-4↑ and eNOS↑; mRNA and protein: miR-133a↓ and GTPCH-1↑	Yin et al. (2019)
db/db (male)	DR	200 mg/kg, i.p., for 10 weeks	FBG↓ and glycogen accumulation↓; serum: TG↓ and AST↓; protein: TNF-α↓, IL-1β↓, HIF-1α↓, VEGF↓, VEGFR-2↓, and NF-κB p65↓	Yin et al. (2021)
SD (male, 6 w, 200 g)	65 mg/kg STZ	100 and 200 mg/kg, i.g., for 8 weeks	Retinal ganglion cell apoptosis↓; protein: MDA↓, ROS↓, SOD↑, CAT↑, GSH↑, p-IκB (Ser32)↓, and NF-κB (nuclear)↓	Zhai et al. (2020)
SD (male, 200–220 g)	55 mg/kg STZ, ip	200 mg/kg, i.g., for 2 weeks	Superior mesenteric artery: NO↑ and contractile responses with L-NAME↑; iliac artery: contractile responses to EFS with phentolamin↓	Zhao et al. (2019b)
SD (male, 120–150 g)	HFD and STZ (30 mg/kg, once, ip)	200 mg/kg, i.g., for 4 weeks	FBG↓ and mesenteric artery vasodilatation↑	Geng et al. (2016)
C57bl/6 (male, 23 ± 2 g)	200 mg/kg alloxan, ip	300 mg/kg, i.g., for 12 weeks	FBG↓, kidney weight↓, BUN↓, serum creatinine↓, and urine protein↓; protein: NF-κB p65↓, ICAM-1↓, TGF-β1↓, fibronectin↓, and IκB-α↑	Liu et al. (2010a)
SD (male, 180 ± 20 g)	35 mg/kg STZ, ip	50, 100, and 200 mg/kg, i.g., for 8 weeks	FBG↓, CCr↓, BUN↓, and Scr↓; protein: ICAM-1↓, VCAM-1↓, and β-arrestin-1/2↑	Tang et al. (2016)
*In vitro*				
Rat mesangial cells	100 ng/ml LPS for 24 h	10, 30, and 90 μM, for 36 h	Protein: p65 (nucleus)↓, ICAM-1↓, TGF-β1↓, iNOS↓, fibronectin↓, p65 (cytoplasm)↑, and IκB-α (cytoplasm)↑	Jiang et al. (2011)
Human platelet	50 μM high glucose	1, 10, 25, and 50 μM, for 90 min	Platelet aggregation↓ and apoptosis↓, NOX↓, ROS↓, superoxide↓, H_2_O_2_↓, intracellular calcium↓, dense granule (ATP)↓, peroxidized cardiolipin↓, and MPTP formation↓; protein: p-ERK↓, PI3K↓, p-p38↓, p-p53↓, Bax↓, Bcl-xl↓, cyto-c↓, and cleaved caspase-3/9↓	Paul et al., 2019
RBMVECs	30 mM glucose for 7 days and 1% O_2_ for 24 h	30 μM	Cell proliferation↑ and migration↑; protein: DPP-4↓, VEGF↑, eNOS↑, HIF-1α↑, and SIRT-1↑	Mi et al. (2019)
Müller cells	33.3 mM glucose for 48 h	20 μM, for 48 h	Cell apoptosis↓ and viability↑; protein: MDA↓, ROS↓, cyto-c↓, cleaved caspase-3/9↓, Bax↓, p-IκB (Ser32)↓, NF-κB (nuclear)↓. GSH↑, SOD↑, CAT↑, GSH↑, and Bcl-2↑	Zhai et al. (2020)
Müller cells	30 and 60 mM glucose for 24 or 48 h	2.5, 5, 10, and 20 μM, for 48 h	Cell viability↑; protein: p-AMPK↑, p-mTOR↓, Bax↓, and Bcl-2↑	Chen et al. (2018)
Mouse MS1 islet microEC of ATCC	45 mM glucose and 31 FU/mL AGEs	2.5, 10, and 40 mg/L, for 24 h	Formation of AGEs↓, NO↑, and NOS↑; mRNA and protein: thrombomodulin↑	Hao et al. (2011)
human artery endothelial cells	25 mM glucose	50 μM, for 1 h	Cell viability↑; protein: p-Akt↑, p-eNOS↑, and p-AMPK↑	Geng et al. (2016)

(Increase, ↑; Decrease, ↓). Abbreviations: 5-HT, 5-hydroxytryptamine; AGEs, advanced glycation end products; AR, aldose reductase; AST, aspartate aminotransferase; Bax, Bcl-2 associated X protein; Bcl-2, B-cell lymphoma 2; BH-4, tetrahydrobiopterin; BK_Ca_, Ca^2+^-activated K^+^ channel; BUN, blood urea nitrogen; CAT, catalase; CCr, creatinine clearance rate; DPP-4, dipeptidyl peptidase 4; EFS, electric field stimulation; eNOS, endothelial nitric oxide synthase; FBG, fasting blood glucose; GR, glutathione reductase; GSH, glutathione; HIF-1α, hypoxia-inducible factor 1α; ICAM-1, intercellular adhesion molecule 1; IKK-β, IkappaB kinase β; IL-1β, interleukin 1β; IL-6, interleukin 6; JNK, c-Jun N-terminal kinase; l-NAME, N^ω^-nitro-l-arginine methyl ester hydrochloride; MCP-1, monocyte chemoattractant protein 1; MDA, malondialdehyde; NF-κB, nuclear factor κB; eIF-2α, eukaryotic initiation factor 2α; NOX, NADPH oxidase; PCA, posterior cerebral artery; ROS, reactive oxygen species; Scr, serum creatinine; SIRT-1, silent information regulator 1; SOD, superoxide dismutase; TG, triglyceride; TNF-α, tumor necrosis factor α; VCAM-1, vascular cell adhesion molecule 1; VEGF, vascular endothelial growth factor.

#### Diabetic Retinopathy

Diabetic retinopathy (DR) is a retinal microvascular disease caused by chronic hyperglycemia leading to angiogenesis in retina (Ai et al., 2019). In recent years, improvement of disordered glucolipid metabolism is considered one of the most effective strategies for the treatment of DM and its complications (Zhao et al., 2021b). Berberine has been shown to block DR development by modulating the glucolipid metabolism and inhibiting the HIF-1α/VEGF/NF-κB pathway (Yin et al., 2021). The pathological processes involved in DR may be related to increased levels of pro-inflammatory factors, leading to oxidative stress and the apoptotic cascade (Ai et al., 2020; Robles-Rivera et al., 2020; Gong et al., 2021). Berberine may deactivate the NF-κB pathway, thus suppressing oxidative stress and cell apoptosis in DR (Zhai et al., 2020). As a major energy receptor and metabolic regulator, AMPK is one of the therapeutic targets for metabolic and vascular diseases (Lu et al., 2019). 10 or 20 μM berberine was shown to have therapeutic effects, protecting Müller cells from 30 mM glucose-induced apoptosis by enhancing autophagy and activating the AMPK/mTOR pathway in DR (Chen et al., 2018). These results together indicate that berberine could attenuate the pathogenesis of DR, mainly through regulating the HIF-1α/VEGF/NF-κB and AMPK/mTOR pathways to inhibit microvascular proliferation, oxidative stress and apoptosis (Table 5).

#### Diabetic Nephropathy

Diabetic nephropathy (DN), one of the most serious microvascular complications of DM, is the leading cause of end-stage renal failure (Lai et al., 2018). The early stage of DN is mainly characterized by abnormal renal hemodynamics, which mainly manifests as decreased vascular resistance in the glomerulus (Toledo et al., 2015). Research has shown that accumulation of ECM production in the glomerular mesangial membrane may be related to NF-κB pathway (Gong et al., 2020). The ameliorative effects of berberine (300 mg/kg) on ECM accumulation may be due to decreased TGF-β1 and ICAM-1 resulting from inhibition of the NF-κB pathway, as shown in a rat model of DN (Liu et al., 2010a). Recent studies have shown that abnormal levels of β-arrestins, including β-arrestins 1 and 2, have a role in microvascular permeability by regulating the production and function of ICAM-1 and vascular cell adhesion molecule 1 (VCAM-1) in the kidneys of a rat model of DN (Noh et al., 2017; Zhang et al., 2018). Orally administration of 100 or 200 mg/kg berberine had renoprotective effects owing to decreased ICAM-1 and VCAM-1 levels and increased β-arrestin 1 and 2 in kidneys of a rat model of DN (Tang et al., 2016). Additionally, inflammation in the kidney is another aggravating factor renal vascular damage in DN (Moreno et al., 2018). Berberine may attenuate LPS-induced inflammation and extracellular matrix accumulation via the NF-κB signaling pathway (Jiang et al., 2011). Given its robust antihyperglycemic and anti-inflammatory activities, and its inhibitory effect on angiogenesis, berberine should be considered a candidate drug for DN (Table 5).

### Intestinal Vascular Diseases

The intestinal mucosal microvasculature is located underneath the intestinal epithelial layer and accurately regulates the passage of molecules across the gut-vascular barrier (GVB) (Zhong et al., 2021). The rat cecal ligation and puncture (CLP) sepsis model was orally treated with berberine (25 and 50 mg/kg for 5 days) showed a protective effect on GVB function in sepsis through the reduction of gut vascular permeability and the suppression of WNT/β-catenin pathways (He et al., 2018). High-density lipoprotein (HDL) particles are related to apoprotein M (ApoM) which is the main carrier of plasma sphingosine-1-phosphate (S1P) (Tavernier et al., 2020). Though only a small proportion of HDL contains ApoM, ApoM-bound S1P is important in maintaining vascular integrity and inhibiting vascular inflammation (Galvani et al., 2015). In a model of polymicrobial sepsis, berberine removed damaged GVB resulting from TLR4-mediated hyperglycemia, insulin resistance and proinflammatory molecule production, thus enriching ApoM gene expression and plasma ApoM via activating the ApoM/S1P pathway (Li et al., 2020b). A previous study showed that the neonatal small intestine is prone to necrotizing enterocolitis (NEC), a severe acquired disease characterized by inflammation (MohanKumar et al., 2019). However, these changes were notably reversed by treatment with berberine; the anti-inflammatory mechanism of berberine in this context may act via suppression of the PI3K/Akt pathway (Fang et al., 2018). Peritoneal adhesions are fibrous tissues that tether organs to one another or to the peritoneal wall and are a major cause of postsurgical morbidity (Tsai et al., 2018). In the normal healing process, ECM can be completely degraded by the proenzyme MMP (Altoé et al., 2021). Berberine prevented adhesion reformation, promoting the activation of MMP-3 and MMP-8 by directly blocking tissue inhibitor of metalloproteinase-1 (TIMP-1) activation in fibroblasts (Liu et al., 2020). To summarize, berberine can inhibit endothelial and gut vascular permeability, inflammation and adhesion reformation in intestine; it may act through several pathways, including the WNT/β-catenin, ApoM/S1P and PI3K/Akt pathway (Table 6).

**TABLE 6:** The *in vivo* and *in vitro* mechanism of berberine in the treatment of intestinal vascular diseases.

Subjects	Model	Doses/Duration	Effects/Mechanisms	References
*In vivo*				
Long-evans rats (male, 270–300 g)	CLP model	25 and 50 mg/kg, i.g., for 5 days	Mortality↓; serum: endotoxin concentration↓; mucosa: microvascular permeability↓	He et al. (2018)
Wistar (male, 260–300 g)	25, 50, and 100 μg/kg ApoM (iv), for 3 days prior to CLP	25, 50, and 100 mg/kg, i.g., for 5 days before CLP	GVB hyperpermeability↓ and mortality↓; plasma: hyperglycemia↓, TNF-α↓, IL-1β↓, insulin resistance↓, and ApoM↑; liver: gluconeogenesis↓; mRNA: PEPCK↓ and ApoM↑	Li et al. (2020b)
C57BL/6JCnc mice (male, postnatal 4-day-old, 5–10 g)	NEC model	5 mg/ml, i.g., for 10 days	Body weight↑ and food intake↑; serum: MD-2↓, TNF-α↓, IL-6↓, Cxcl-1↓, TLR-4↓, and NF-κB↓	Fang et al. (2018)
Wistar (male, 200–220 g)	Primary adhesion rat model	1.5 mg/ml, i.p., for 2.0 ml	Peritoneal adhesion↓, cicatricial adhesion reformation↓, vascular proliferation↓, fibrin and collagen deposition↓; mRNA: collagens 1/3↓; protein: VEGF-α↓, TIMP↓, and MMP-3/8↑	Liu et al. (2020)
*In vivo*				
RIMECs	50 ng/ml LPS	10 and 20 nM	Transendothelial permeability↓ and TEER↑; protein: β-catenin↑, claudin-12↑, and VE-cadherin↑	He et al. (2018)
HepG2	100 ng/ml LPS	5, 10, and 20 nM	mRNA: TLR-4↓	Li et al. (2020b)
Epithelial cells from the small intestine	5% CO2 humidified atmosphere at 37°C	5 mg/ml, for 24 h	Cell apoptosis↓ and viability↑; protein: caspase-3/9↓, p-PI3K↓, PI3K↓, Akt↓, p-Akt↓, survivin↑, cyto-c↑, c-Myc↑, p53↑, IFN-γ↑, Bcl-2↑, and EGF↑	Fang et al. (2018)

(Increase, ↑; Decrease, ↓). Abbreviations: Akt, protein kinase B; ApoM, apoprotein M; Bcl-2, B-cell lymphoma 2; CLP, cecal ligation and puncture; c-Myc, Myc proto-oncogene protein; Cxcl-1, chemokine (C-X-C motif) ligand 1; Cyto-c, cytochrome c; EGF, epithelial growth factor; GVB, gut-vascular barrier; IFN-γ, interferon γ; IL-1β, interleukin 1β; MD-2, myeloid differentiation protein 2; MMP-3/8, matrix metalloprotease 3/8; NF-κB, nuclear factor κB; PI3K, phosphoinositide 3-kinase; TEER, transendothelial electrical resistance; TIMP-1, tissue inhibitor of metalloproteinase 1; TLR-4, toll-like receptor 4; TNF-α, tumor necrosis factor α; VE-cadherin, vascular endothelial cadherin; VEGF-α, vascular endothelial growth factor α.

### Vasculature in Cancer

Angiogenesis is a process in which new blood vessels form and grow from pre-existing vessels; this process occurs under healthy and pathological conditions, such as cancer (Fang et al., 2021). VEGF promotes angiogenesis in endothelial cells. Berberine has been shown to suppress angiogenic action, HUVEC proliferation and migration, by inhibiting VEGF, against cancerous Meth A cells and hepatocellular carcinoma (Jie et al., 2011; Yahuafai et al., 2018). VEGF-2 is a major mediator of the biological effects of VEGF, and therefore plays an important role in tumor angiogenesis (Sharaky et al., 2020). Berberine was shown to inhibit angiogenesis in glioblastoma xenografts by targeting the VEGFR-2/ERK pathway (Jin et al., 2018). In tumor development, chronic inflammation leads to a sharp increase in VEGF expression (Crusz and Balkwill, 2015). Elevated inflammatory cytokine levels and epithelial–mesenchymal transition in glioma cells were shown to be reversed by 100 μM berberine; the mechanism for this may be via suppressing ERK-1/2 signaling and production of IL-1β and IL-18 (Tong et al., 2019). VEGFR-3 in lymphatic endothelial cells were essential for the development of VSMCs (Antila et al., 2017). Clinically, elevated levels of VEGFR-3 are thought to be correlated with cancers resulting from metastasis via the lymph nodes, such as renal carcinoma (Guida et al., 2014). Following an inflammatory stimulus, the production of ROS by oxidative stress and energy deficit at mitochondrial, lysosomal, or ER loci can directly lead to irreversible damage of tumor cells and initiate cell apoptosis and autophagy (Reuter et al., 2010). Combined with photodynamic therapy, 20 μM berberine triggered metabolite changes in renal carcinoma cells, resulting in inhibited cell proliferation, tumorigenesis, and angiogenesis, and inducing autophagy and apoptosis via increased ROS generation (Lopes et al., 2020). Another study implied that 12-O-tetradecanoyl phorbol-13-acetate (TPA) significantly increased the level of VEGF and fibronectin in both MCF7 and T47D breast cancer cells (Kim et al., 2009). TPA-induced VEGF and fibronectin expression was decreased by berberine treatment, via inhibition of the PI3K/A pathway, in breast cancer cells (Kim et al., 2013). Taken together, these results show that berberine may effectively inhibit the proliferation and angiogenesis of tumor cells by inhibiting VEGF, ERK and PI3K/Akt pathways and promote the apoptosis of tumor cells (Table 7).

**TABLE 7:** The *in vivo* and *in vitro* mechanism of berberine in the treatment of vasculature in cancer.

Subjects	Model	Doses/Duration	Effects/Mechanisms	References
*In vivo*				
BALB/c (male, 5 w, 18–22 g)	Meth A sarcoma-bearing mice	5 mg/kg, i.p., for 23 days	Tumor volume↓	Yahuafai et al. (2018)
Athymic nude mice (5–6 w)	Ectopic and orthotopic xenograft model	50 mg/kg, i.g., for 4 weeks	Tumor volume↓ and vascular density↓; protein: p-VEGFR2↓, p-p38↓, and p-ERK↓	Jin et al. (2018)
*In vitro*				
HUVECs, Meth A murine sarcoma cells	NA	12.5, 25, and 50 μg/ml, for 24 h	Cell viability↓; protein: VEGF↓	Yahuafai et al. (2018)
The HCC cell line HepG-2 and HUVECs	NA	5, 10, and 15 μM, for 24 h	Cell proliferation↓, cell migration↓, and tube formation↓; mRNA and protein: VEGF↓	Jie et al. (2011)
U87 and U251 human glioblastoma cell lines huvec	NA	6.25, 12.5, 25, 50, 100, and 200 μM, for 48 h	U87 and U251: cell viability↓ and proliferation↓; huvec: cell migration↓ and tube formation↓	Jin et al. (2018)
Human U87 and U251 cell lines; oligodendrocytes	NA	50 and 100 μM, for 24 h	Cell viability↓ and migration↓; protein: vimentin↓, α-SMA↓, p-ERK↓, α-catenin↑, and β-catenin↑; mRNA and protein: IL-1β↓, IL-18↓, and caspase-1↓	Tong et al. (2019)
Human RCC cell lines and human renal tubular epithelial cells	NA	20 μM, for 24 h	Associated with PDT: cell viability↓, lactate↓, phototoxicity↑, ROS↑, lysine↑, and autophagy↑; mRNA: TERT↓cyt and PLK-3↑; protein: caspase-3↑	Lopes et al. (2020)
MCF-7 and T47D human breast cancer cells	10 nM TPA for 24 h	100 μM, for 1 h	VEGF↓ and fibronectin↓	Kim et al. (2013)

(Increase, ↑; Decrease, ↓). Abbreviations: AP-1, activator protein 1; Bax, Bcl-2 associated X protein; Bcl-2, B-cell lymphoma 2; ERK, extracellular signal-regulated kinase; HUVECs, human umbilical vein endothelial cells; IL-1β, interleukin 1β; JAK-2, janus kinase 2; MMP-2, matrix metalloprotease 2; PLK-3, polo-like kinase 3; ROS, reactive oxygen species; TERT, telomerase reverse transcriptase; VEGFR-2, vascular endothelial growth factor receptor 2; α-SMA, α-smooth muscle actin.

### Other Vascular Diseases

Rheumatoid arthritis (RA) is a chronic inflammatory autoimmune disease that may involve angiogenesis, particularly during the earliest stages of the disease (Yang et al., 2018; Lyu et al., 2021). Angiogenesis is strictly regulated by several pro- and antiangiogenic factors including VEGF, which have been suggested to be involved in neovascularization in RA joints (Li et al., 2018a). Berberine was shown to have anti-inflammatory and antiangiogenic effects in a rat model of RA by decreasing the level of inflammatory factors, and suppressing p-ERK, p-p38 and p-JNK activation (Wang et al., 2014). Additionally, it is reported that doxorubicin-induced vascular congestion and inflammatory cell infiltration in the liver were largely attenuated by berberine pretreatment (Zhao et al., 2012).

## Conclusion and Perspectives

**FIGURE 5:**
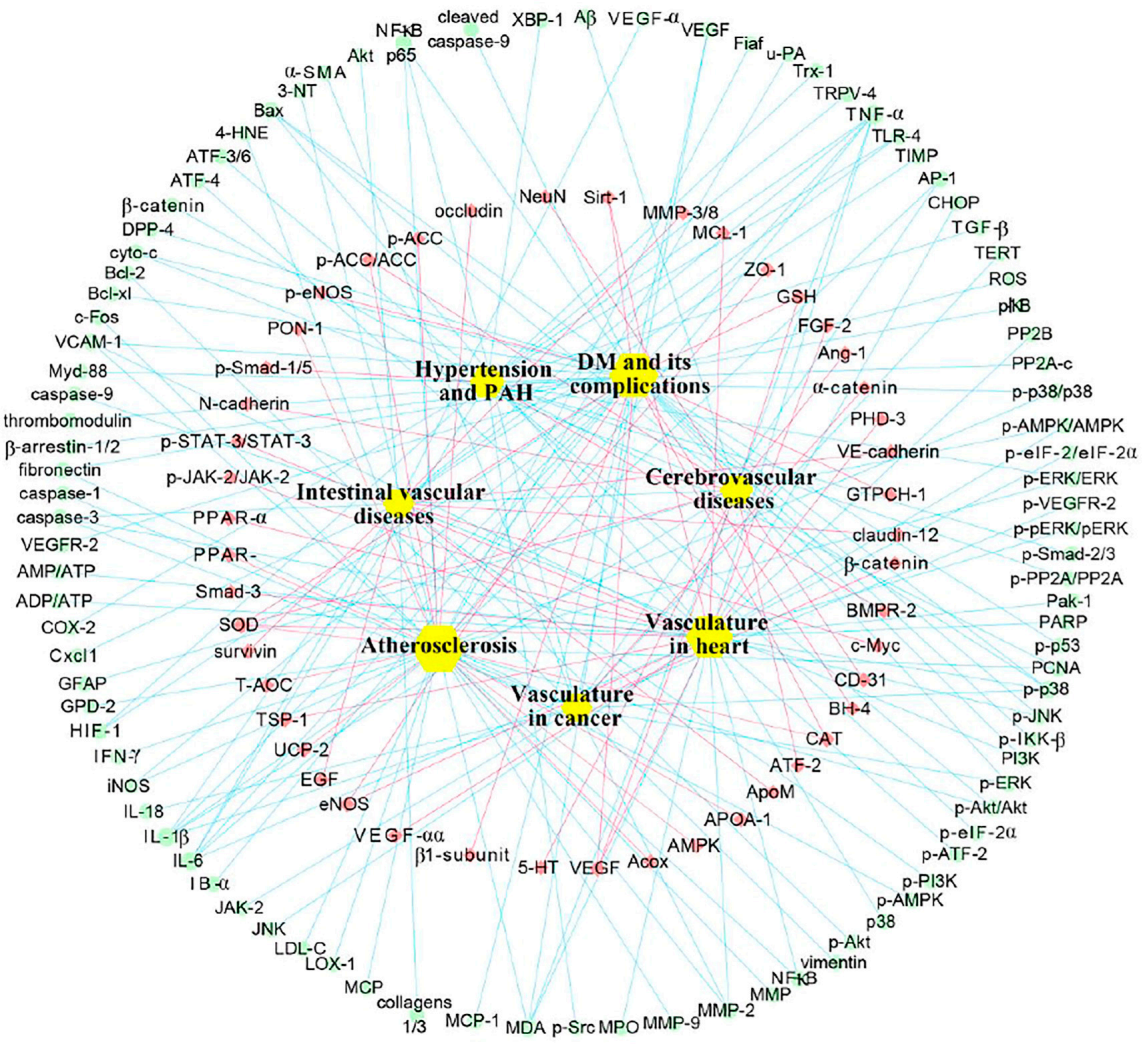
Network diagram of berberine involved gene and protein targets in treating vascular diseases. The yellow cubes in the innermost circle are the vascular disease treated with berberine, the red dots in the middle circle represent the up-regulated gene and protein targets, and the blue dots in the outside circle denote the down-regulated gene and proteins. Abbreviations: 3-NT, 3-nitrotyrosine; 4-HNE, 4-hydroxynoneal; 5-HT, 5-hydroxytryptamine; ADP, adenosine diphosphate; Akt, protein kinase B; AMP, adenosine monophosphate; AMPK, AMP-activated protein kinase; AP-1, activator protein 1; APOA-1, apolipoprotein A-1; ApoM, apoprotein M; ATF-4, activating transcription factor 4; ATP, adenosine triphosphate; Bax, Bcl-2 associated X protein; Bcl-2, B-cell lymphoma 2; BH-4, tetrahydrobiopterin; BMPR-2, bone morphogenetic protein type 2; CAT, catalase; CD-31, platelet endothelial cell adhesion molecule 1; CHOP, C/EBP homologous protein; CLP, cecal ligation and puncture; c-Myc, Myc proto-oncogene protein; COX-2, cyclo-oxygenase 2; CPT-1α, carnitine palmitoyl transferase 1α; Cyto-c, cytochrome c; DM, diabetes mellitus; DPP-4, dipeptidyl peptidase 4; EGF, epithelial growth factor; eIF-2α, eukaryotic initiation factor 2α; eNOS, endothelial nitric oxide synthase; ERK, extracellular signal-regulated kinase; FABP-4, fatty acid binding protein 4; FGF-2, fibroblast growth factor 2; GFAP, glial fibrillary acidic protein; GPD-2, glycerol-3-phospate dehydrogenase 2; GSH, glutathione; HIF-1α, hypoxia-inducible factor 1α; IFN-γ, interferon γ; IKK-β, IkappaB kinase β; IL-6, interleukin 6; iNOS, inducible nitric oxide synthase; JNK, c-Jun N-terminal kinase; LDL-c, low density lipoprotein cholesterol; LOX-1, low-density lipoprotein receptor 1; MAO, monoamine oxidase; MCL-1, myeloid cell leukemia 1; MCP, monocyte chemoattractant protein; MDA, malondialdehyde; MMP-2, matrix metalloprotease 2; MPO, myeloperoxidase; Myd-88, myeloid differentiation factor 88; NF-κB, nuclear factor κB; PAH, pulmonary arterial hypertension; Pak-1, p21-activated kinase 1; PARP, poly (ADP-ribose) polymerase; PCNA, proliferating cell nuclear antigen; PHD-3, prolyl hydroxylase 3; PI3K, phosphoinositide 3-kinase; PON-1, paraoxonase 1; PP2Ac, protein phosphatase 2Ac; PP2B, calcineurin; PPAR-α, peroxisome proliferator-activated receptor α; ROS, reactive oxygen species; SIRT-1, silent information regulator 1; Smad-3, small mother against decapentaplegic 3; SOD, superoxide dismutase; STAT-3, signal transducer and activator of transcription 3; T-AOC, total antioxidant capacity; TERT, telomerase reverse transcriptase; TGF-β, transforming growth factor β; TIMP, tissue inhibitor of metalloproteinase; TLR-4, toll-like receptor 4; TNF-α, tumor necrosis factor α; TRPV-4, transient receptor potential vanilloid 4; Trx-1, Thioredoxin 1; TSP-1, thrombospondin 1; UCP-2, uncoupling protein 2; u-PA, urokinase-type plasminogen activator; VCAM-1, vascular cell adhesion molecule 1; VEGF-α, vascular endothelial growth factor α; XBP-1, X-box binding protein 1; ZO-1, zona occluden 1; α-SMA, α-smooth muscle actin.

Berberine is a multifunctional, natural product with therapeutic potential in vascular diseases, including cardiovascular disease, atherosclerosis, hypertension, cerebrovascular disease, diabetes and associated complications, intestinal vascular disease and cancer (Zhu et al., 2018; Martini et al., 2020; Yin et al., 2021). The present review has shown the excellent protective effect of berberine in diverse vascular diseases by preserving vascular endothelial cells, improving vascular remodeling and vasoconstriction, and suppressing inflammation, oxidative stress, autophagy, and apoptosis, based on recent *in vitro* and *in vivo* experimental reports (Figure 2). Moreover, berberine also modulates the concentration of Na^+^, Ca^2+^ and lipid metabolism in the VSMCs (Table 4). What is more, the network diagram of “vascular diseases-target-pathways” was shown in Figure 5, and the underlying mechanism of berberine in the treatment of vascular diseases mentioned in this paper may be related to multiple pathways, including HIF-1α/VEGF, STAT, MAPK, NF-κB, SIRT, PI3K/Akt, AMPK and TRPV-4.

Pharmacokinetics is principally to quantitatively assess the absorption, distribution, metabolism, and excretion (ADME) properties of drugs within a living organism that determine the safety and effective of drugs. Berberine is widely distributed in multiple tissues and organs after entering circulation, and can still accumulate in plasma despite keeping a low-rise concentration (Han et al., 2021a; Chen et al., 2021). According to our review, there are still many issues to overcome regarding the use of berberine to treat the vascular diseases. First, the bioavailability of orally-administered berberine *in vivo* is low due to first-pass elimination. It is therefore imperative to investigate alternative modes and methods of drug delivery with the aim of increasing the bioavailability of berberine. Numerous studies have showed that the strategic use of nanotechnology, including nanocarriers, liposomes, and microfluidic technology-assisted preparation methods, may increase the bioavailability of berberine for use in cardiovascular and metabolic diseases (Allijn et al., 2017; Guo et al., 2019b; Dewanjee et al., 2020). Structural modification of berberine may also improve bioavailability and efficacy and reduce adverse drug reactions (Mbese et al., 2019; Luo et al., 2021). Han et al. (2019); synthesized, water-soluble berberine derivatives with modified 9-O-monosaccharide (administered at concentrations of 0.2, 1 and 5 μg/ml) were shown to have antidiabetic effects, with lower cytotoxicity and a half-maximal inhibitory concentration (IC_50_) nearly 1.5 times than that of unmodified berberine in HepG2 liver cancer cells. Additionally, this review found that the toxicity of berberine and its derivatives have been rarely investigated. Considering the long-term development for berberine prevention and treatment of vascular diseases, comprehensive toxicity investigations, especially potentially cumulative toxicity *in vivo* studies, need to be carried out. It is also necessary to explain the efficacy and toxicity of berberine for use in human pharmacokinetic studies, and the identification of the ideal dosage are of enormous significance if side effects associated with drug accumulation are to be avoided. Relatively newly developed biological techniques, including microfluidic technology, computational toxicological methods, hepatoid cell models, and high-throughput chip models, could be employed to explore the toxicity of berberine (Banerjee et al., 2016; Kimura et al., 2018). In addition, single-cell *in vitro* models, as reported in the literature reviewed here, may not adequately reflect the pathogenesis of diseases *in vivo*. A multi-organ *in vitro* model based on microfluidic technology may be more helpful models of vascular diseases for the evaluation of the safety and efficacy of berberine (Rothbauer et al., 2018; Malik et al., 2021). For the clinical trial of berberine on vascular disease, many factors limit its clinical application, including the low methodological quality and drug–drug interactions (Imenshahidi and Hosseinzadeh, 2019). Therefore, the development of a standardized dosage, administration route, duration and adverse reaction of berberine could also be pursued in clinical settings for better therapeutic efficacy and safety.

Concurrently, we should strengthen the mechanism of berberine in the treatment of vascular diseases. Novel technologies that could be employed to explore the mechanism of action of berberine in the treatment of vascular diseases include CRISPR–CAS-9 gene editing, metabolomics, proteomics, and genomics. Most importantly, recently published investigations of the pharmacological mechanisms of berberine may provide new insights into the treatment of vascular diseases by berberine. For example, Zhao and others demonstrated that 560 mg/kg berberine administered orally in *Coptis chinensis* can significantly increase insulin secretion via the potassium voltage-gated channel subfamily H member 6 (KCNH-6) potassium channel in mice with HFD-induced hyperglycemia (Zhao et al., 2021a). Overall, this study comprehensively reviewed and summarized the pharmacokinetics properties and therapeutic potentials of berberine in diverse vascular diseases, thus providing experimental evidence for future research to discover novel drugs from Chinese medicine monomers.
